# Benchmarking predictive methods for small-angle X-ray scattering from atomic coordinates of proteins using maximum likelihood consensus data

**DOI:** 10.1107/S205225252400486X

**Published:** 2024-07-10

**Authors:** Jill Trewhella, Patrice Vachette, Andreas Haahr Larsen

**Affiliations:** a School of Life and Environmental Sciences University of Sydney NSW2006 Australia; b Institute for Integrative Biology of the Cell (12BC) Université Paris-Saclay, CEA, CNRS Gif-sur-Yvette Paris91198 France; c Department of Neuroscience University of Copenhagen Blegdamsvej 3 2200Copenhagen Denmark; University of Auckland, New Zealand

**Keywords:** biomolecular small-angle scattering, SAXS, SAXS profile modelling, hydration layer, waters of hydration, consensus data, benchmarking, molecular dynamics, simulation, maximum likelihood

## Abstract

Stimulated by informal conversations at the XVII International Small Angle Scattering (SAS) conference (Traverse City, 2017), an international team of experts undertook a round-robin exercise to produce a large dataset from proteins under standard solution conditions. These data were used to generate consensus SAS profiles for xylose isomerase, urate oxidase, xylanase, lysozyme and ribonuclease A. Here, we apply a new protocol using maximum likelihood with a larger number of the contributed datasets to generate improved consensus profiles. We investigate the fits of these profiles to predicted profiles from atomic coordinates that incorporate different models to account for the contribution to the scattering of water molecules of hydration surrounding proteins in solution. Programs using an implicit, shell-type hydration layer generally optimize fits to experimental data with the aid of two parameters that adjust the volume of the bulk solvent excluded by the protein and the contrast of the hydration layer. For these models, we found the error-weighted residual differences between the model and the experiment generally reflected the subsidiary maxima and minima in the consensus profiles that are determined by the size of the protein plus the hydration layer. By comparison, all-atom solute and solvent molecular dynamics (MD) simulations are without the benefit of adjustable parameters and, nonetheless, they yielded at least equally good fits with residual differences that are less reflective of the structure in the consensus profile. Further, where MD simulations accounted for the precise solvent composition of the experiment, specifically the inclusion of ions, the modelled radius of gyration values were significantly closer to the experiment. The power of adjustable parameters to mask real differences between a model and the structure present in solution is demonstrated by the results for the conformationally dynamic ribonuclease A and calculations with pseudo-experimental data. This study shows that, while methods invoking an implicit hydration layer have the unequivocal advantage of speed, care is needed to understand the influence of the adjustable parameters. All-atom solute and solvent MD simulations are slower but are less susceptible to false positives, and can account for thermal fluctuations in atomic positions, and more accurately represent the water molecules of hydration that contribute to the scattering profile.

## Abstract 2

Consensus small-angle X-ray scattering (SAXS) data from five proteins in solution, generated from 171 independent measurements on 12 beamlines using a maximum likelihood method, are used to benchmark computational methods for predicting SAXS profiles from atomic coordinates. The results reveal important strengths and limitations of different methods that are serving a growing community of users in applications ranging from fundamental integrative structural biology to drug discovery and development.

## 1. Introduction

**Figure 1:**
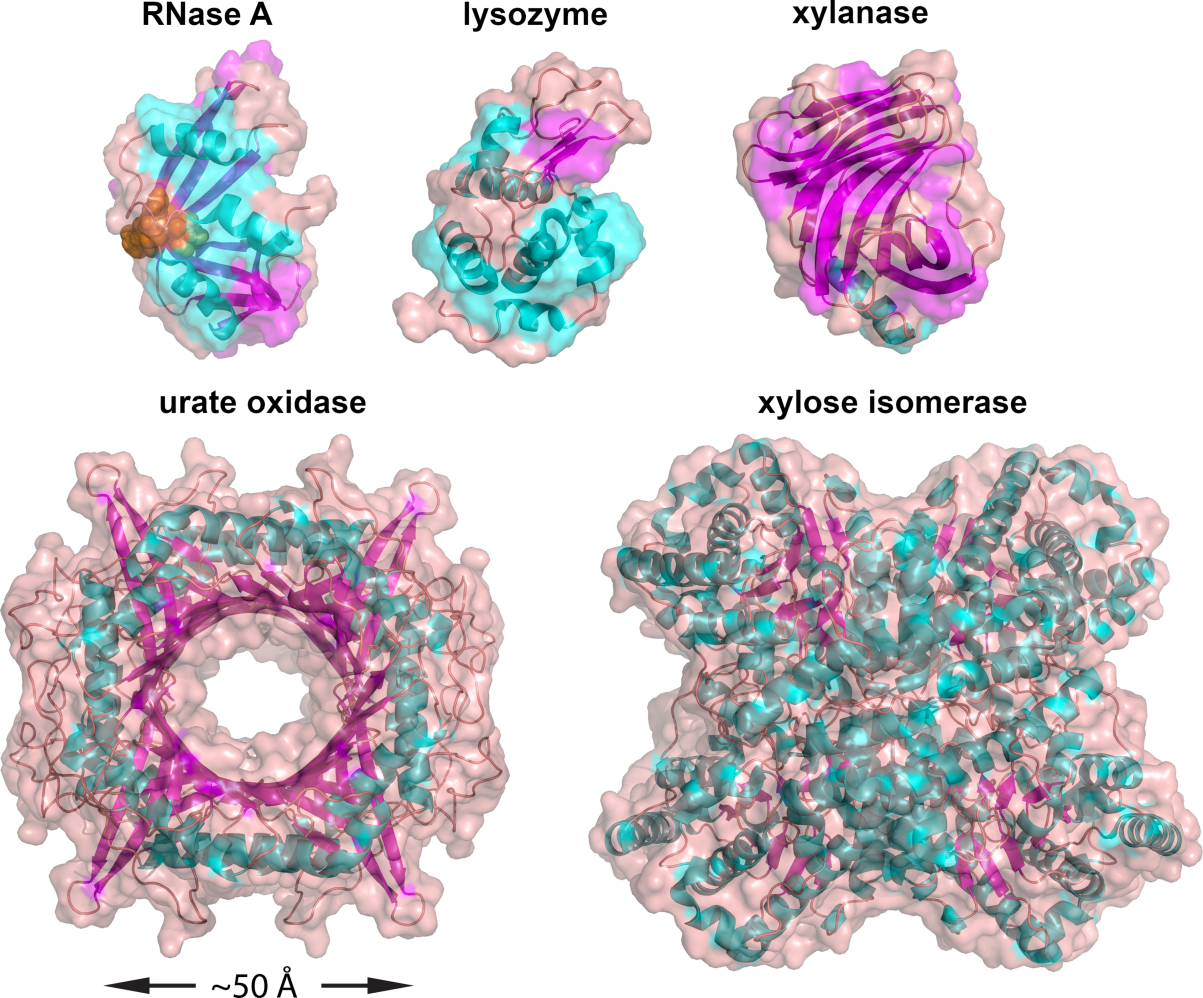
Crystal structures for the five proteins of this study represented as ribbons with colour-coded secondary structures (helices: cyan; β-strands: magenta; other: orange) with a semi-transparent surface (orange) overlay. The PDB entry, resolution and molecular mass from chemical composition for each protein is: RNase A, 7rsa, 1.26 Å, 13.690 kDa; lysozyme, 2vb1, 0.65 Å, 14.313 kDa; xylanase, 2dfc, 1.19 Å, 20.825 kDa; urate oxidase tetramer, 3l8w, 1.00 Å, 136.603 kDa; xylose isomerase tetramer, 1mnz, 0.99 Å, 172.910 kDa, respectively. The urate oxidase tetramer structure includes an added C-terminal SLKSKL missing from the crystal structure, whereas the xylose isomerase includes a missing N-terminal me­thio­nine. Amino acids 47–48, 80–81 and 102–104 in RNase A, shown as orange spheres, form the hinge at the base of the V shape of the three β-strands that facilitates subtle domain dynamics required for RNase A activity.

In 2019, members of the international small-angle scattering (SAS) community conducted a round-robin exercise (Trewhella *et al.*, 2022 ▸) referred to hereafter as the ‘round-robin study’, in which 247 SAS profiles, including 171 small-angle X-ray scattering (SAXS) profiles from 12 beamlines and 76 small-angle neutron scattering (SANS) profiles from four beamlines, were collected from a set of five proteins. The specific objectives were to (1) measure proteins with known structures at different beamlines using a common source for each protein and standard buffers, (2) compare datasets from each protein for consistency and (3) make a set of consensus profiles to benchmark methods for calculating scattering profiles from atomic coordinates available to the research community. The protein structures selected for this study were all globular with high-resolution crystal structures (0.65–1.26 Å), representing a range of sizes (14–173 kDa) with different secondary structure compositions, and the urate oxidase tetramer served as an example of a structure with a central solvent channel (Fig. 1 ▸). Careful merging of data from the same instrument measured using in-line SEC- and batch-modes, or low- and high-concentration data from batch measurements, was generally successful in eliminating small amounts of aggregate or interparticle interference from the scattering in the low-*q* regime. The combined consensus profiles provided substantially improved statistical precision up to *q* = 1 Å^−1^ for SAXS data compared with individual measurements. Overall, the SAS data were shown to be reproducible, albeit requiring a multiplicative factor to scale to the experimental data, as well as an additive constant to correct for uncertainties in background scattering and solvent subtraction. Consensus SAXS and SANS profiles for the stable tetramers of xylose isomerase and urate oxidase, and monomers of xylanase, lysozyme and ribonuclease A (RNase A) plus the contributing data for each were deposited in the Small Angle Scattering Biological Data Bank (SASBDB) (Kikhney *et al.*, 2020 ▸; Valentini *et al.*, 2015 ▸). Preliminary comparisons of the consensus profiles with predicted profiles using various popular methods indicated good agreement with their general features, but with some differences evident in error-weighted residual difference plots. Recently, explicit-solvent SAXS/SANS molecular dynamics (MD) calculations with 18 different combinations of protein force fields and water models were used to predict hydration shell contrast for comparison with the combined SAXS/SANS radius of gyration (*R*_g_) values from the consensus data (Linse & Hub, 2023 ▸). Several but not all of the force fields were found to show remarkable agreement with the experiment.

We present here an improved protocol that utilizes more of the original contributed datasets with a maximum likelihood method for combining data that yields updated consensus SAXS profiles consistent with those from the original study, but for three of the five proteins the error distributions are smoother with smaller overall magnitudes. With these updated consensus profiles, we investigate in more detail the residual differences of fits obtained with different computational modelling approaches.

An important distinction among different approaches to modelling SAS profiles is how they account for the water molecules of hydration surrounding a protein in solution, which has been long understood to contribute to the scattering pattern (Zaccai & Jacrot, 1983 ▸), but for which there is relatively limited experimental data. In their combined SAXS and SANS study of lysozyme, *E. coli* thio­redoxin reductase and protein R1 of *E. coli* ribonucleotide reductase, Svergun *et al.* (1998 ▸) concluded that ‘The results point to the existence of a first hydration shell with an average density ≈10% larger than that of the bulk solvent under the conditions studied. Comparisons with the results of other studies suggest that this may be a general property of aqueous interfaces.’ More recent studies have estimated the hydration shell density is generally ∼5% larger than the bulk solvent (*e.g.* Grishaev *et al.*, 2010 ▸; dos Reis *et al.*, 2011 ▸). This concept of a ‘hydration layer’ has guided many in the development of approaches to calculating scattering profiles with an implicit hydration layer assumed to be some sort of uniform scattering density shell. Alternately, the contribution of the water molecules of hydration to the scattering has been accounted for using explicit water models, including via molecular dynamics (MD) simulations. In the context of this study, it is pertinent to briefly review here different approaches to modelling the water molecules of hydration and to define and understand the role of the parameters that will be presented and discussed. Many different SAS modelling programs have been developed, and we cannot comprehensively cover them all here [for recent reviews see the literature (Gerstein & Richards, 2012 ▸; Brosey & Tainer, 2019 ▸; Chatzimagas & Hub, 2022*b* ▸; Trewhella, 2016 ▸; Gräwert & Svergun, 2020 ▸)]. Our focus is on a representative set from what are currently easily accessed and widely used programs that are regularly maintained and updated by their developers.

### 1.1. Implicit hydration-layer models

The first published method for calculating scattering profiles from atomic coordinates that was made broadly available and became widely used was *CRYSOL* (Svergun *et al.*, 1995 ▸). In their notation, the scattering from a biomolecule in solution, *I*(*q*), is expressed as
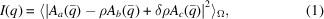
where *q* is the amplitude of the momentum transfer 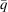. 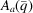 is the scattering amplitude of the biomolecule *in vacuo*; 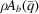 is the missing scattering amplitude due to the bulk solvent (scattering density ρ) in the volume excluded by the bio­molecule (the total excluded volume); and 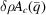 is the scattering amplitude due the hydration layer where δρ = ρ_h_ − ρ is the difference in scattering density of the hydration layer, ρ_h_, with respect to the bulk solvent ρ. The brackets 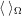 indicate the rotational average that accounts for the random orientation of biomolecules in solution. Since the publication of *CRYSOL*, other developers have accepted the basic notion of a hydration ‘layer’ having a fixed contrast with respect to the bulk solvent as a first-order approximation and have developed various computational approaches to its description. In all these methods, in addition to scaling to the experimental data and having the option for a constant subtraction to account for potential solvent subtraction errors, free parameters relating to the total excluded volume, and the contrast of the hydration layer, δρ, are optimized when fitting the calculated profile to the experiment.

The three examples of programs used here are freely available on web servers for the general user: *CRYSOL* (Svergun *et al.*, 1995 ▸), *Pepsi-SAXS* (Grudinin *et al.*, 2017 ▸) and *FoXS* (Schneidman-Duhovny *et al.*, 2016 ▸). The main differences among these programs are: (i) the use of a multipole expansion to calculate scattering intensities and amplitudes in the spherical coordinate system first introduced by Stuhrmann (1970 ▸) as in *CRYSOL* and *Pepsi-SAXS* versus using the Debye equation (Debye, 1915 ▸) as in *FoXS*, (ii) the precise details of the modelled hydration layer, and (iii) the details with respect to the atomic form factors used.

In all three programs, the total excluded volume is adjusted. The excluded volume per atomic group *i* can be calculated using
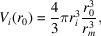
where *r_i_* are the values of the atomic group radii of the protein (Gerstein & Richards (2012 ▸); Tsai *et al.*, 1999 ▸) and *r_m_* is the expected average radius of atomic groups for the entire protein (typically ∼1.62 Å). The ratio *r*_0_/*r_m_* is a scaling factor, which we will refer to as *r*_Sc_, it adjusts the *r_i_* values to give what is referred to as ‘effective’ atomic radii, and the total excluded volume adjustment is *r*_Sc_^3^. The original *CRYSOL* optimized total excluded volume while keeping *r*_Sc_ in the range 0.96 < *r*_Sc_ < 1.04 (Svergun *et al.*, 1995 ▸) and later versions of *CRYSOL* expanded this range (*e.g.* v2.8.4 uses a search range 0.89 ≤ *r*_Sc_ ≤ 1.136). *Pepsi-SAXS* optimizes within the range 0.95 ≤ *r*_Sc_ ≤ 1.05. *FoXS* reports the parameter *c*_1_, also a scaling factor for atomic radii to optimize the total excluded volume [see equation (3)], with the same allowed ranges as *Pepsi-SAXS*, *i.e.* 0.95 ≤ *c*_1_ ≤ 1.05 (Schneidman-Duhovny *et al.*, 2013 ▸).

The hydration layer is treated somewhat differently in each of the programs, and each implementation uses different approaches to speed up calculations. *CRYSOL* defines a continuous hydration shell of fixed thickness (3 Å) with a contrast in the range 0 ≤ δρ ≤ δρ_max_, where δρ_max_ was originally set at 0.06 e Å^−3^ (or ∼18% of the bulk solvent density, default value 0.334 e Å^−3^) based on data from Perkins (1986 ▸). Later versions of *CRYSOL* allowed larger δρ_max_ values (*e.g.* 0.075 e Å^−3^ in v2.8.4, or 22% of the default bulk solvent density). The most recent *CRYSOL* (v3.2.1) has a new option to define the hydration layer as a shell of dummy waters that may be more appropriate for highly flexible structures or irregular shaped structures (Franke *et al.*, 2017 ▸). For the classic directional water layer, the surface of the protein is defined using a Fibonacci grid in polar angle coordinates that determines sampling directions; the greater the order of the Fibonacci grid, *F_n_*, the greater the number of points to define the surface.

*Pepsi-SAXS* generally follows the formalism of *CRYSOL* but with form factors that have been calculated with specific consideration of charged groups. Further, to speed up the calculations a fast model is used for computation of the hydration layer based on a grid of points, with a cell size of 3–4 Å. An algorithm removes cells that overlap with atoms in the biomolecule and cells that are beyond the hydration shell. The assumed thickness of the hydration shell is 3 or 5 Å for biomolecules with *R*_g_ < 15 Å or *R*_g_ > 20 Å, respectively, and a linear interpolation is used to determine the hydration shell thickness for *R*_g_ values in between. The default range for δρ optimization is 0 ≤ δρ ≤ 0.0334 e Å^−3^, *i.e.* a maximum of 10% of the default bulk solvent density (0.334 e Å^−3^).

As noted above, both *CRYSOL* and *Pepsi-SAXS* calculate scattering intensities and amplitudes in the spherical coordinate system. In *CRYSOL*, the default number of harmonics, *L*, for the calculation is 20 and the user can specify *L* up to 100, with larger values recommended for larger or more extended structures and for computing intensities at higher *q* values. Larger values of *L* and *F_n_* require more time for the calculation. *Pepsi-SAXS* speeds up calculations using an adaptive order multipole expansion to determine the value of *L* required based on the *R*_g_ value for the hydration shell and *q*_max_. Expansion coefficients are sampled at 2*L* equidistant points before determining the values at each experimental *q* value using cubic spline interpolation.

*FoXS* uses the Debye formula for computing the SAXS intensity profile (Debye, 1915 ▸):
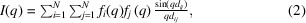
where *d_ij_* is the distance between atoms *i* and *j*, and *N* is the number of atoms. In their implementation, the hydration layer is accounted for within the atomic form factors *f_i_*(*q*):
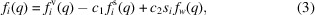
where 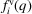 is the atomic form factor *in vacuo*, 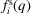 is the form factor of the dummy atom representing the displaced solvent, *s_i_* is the fraction of solvent accessible surface of the atom and *f_w_*(*q*) is the hydration layer water form factor. The computational time for the Debye formula is proportional to square of the number of atoms in the molecule, *N*, times the number of points in the *I*(*q*). To speed up calculations, *FoXS* uses approximations for form factors and distances that reduce the computational time to *N*^2^. The two free parameters are *c*_1_, the scaling factor applied to the form factor for dummy atoms representing the displaced solvent that adjusts the total excluded volume, and *c*_2_ that adjusts the density of the water in the hydration layer atom-by-atom and is related to δρ, but not in a simple way. The allowed range for *c*_2_ is −2 to 4.0 in steps of 0.1. Negative values for *c*_2_ are allowed in *FoXS* with the justification that ‘the density of the hydration layer around the protein can, in principle, be lower than that of bulk water (assigned as 0.334 e Å^−3^), depending on the amount of surface charge’ (Schneidman-Duhovny *et al.*, 2013 ▸). The hydration shell density for *c*_2_ = 4.0 is 0.388 e Å^−3^ and for *c*_2_ = −2.0 is 0.307 e Å^−3^, *i.e.* δρ values of +16% and −8% of the default bulk solvent density, respectively. *Pepsi-SAXS* by comparison has an option to allow for a slightly negative δρ (up to 0.015 e Å^−3^, or −4% of the default bulk solvent density), but it is not the default. For *CRYSOL*, negative δρ is not allowed.

### 1.2. Explicit models for the water layer using molecular dynamics

Early pioneering works for explicit modelling of water molecules of hydration included those by Merzel & Smith (2005 ▸, 2002*a* ▸,*b* ▸) who used multipole expansions (like *CRYSOL*) to compute scattering intensities from MD simulations of lysozyme in water, and Oroguchi *et al.* (2009 ▸) who used MD simulations to predict the scattering profile of restriction endonuclease EcoO109I. However, it was the work by Park *et al.* (2009 ▸) that marked a breakthrough in the utilization of MD simulations in this arena by making use of two short simulations of a pure solvent box and the protein within a solvent box where the protein is associated with a water shell defined by its thickness. Intensities were averaged over the solvent degrees of freedom and over the rotational degrees of freedom of the solute which is ‘frozen’ with no internal motions.

Chen & Hub (2014 ▸) built on this early work to develop the program *WAXSiS* (Wide-Angle X-ray Scattering in Solution) that is available at https://waxsis.uni-saarland.de/about/ (Knight & Hub, 2015 ▸). They also perform two, short (typically a few tens or hundreds of picoseconds) explicit-solvent, all-atom MD simulations of the protein within a solvent box, and a pure solvent box that is used to compute the excluded solvent scattering. All backbone atoms of the protein and heavy atoms of ligands are restrained by a harmonic potential during the simulation to ensure the protein only explores conformations like the initial structure and precludes any large-scale dynamics such as domain movements. With this restriction, the simulation provides an atomic description that accounts for thermal fluctuations of the protein and the solvent, including the waters of hydration and bulk solvent alike. The protein atomic fluctuations are considered to contribute significantly to scattering intensities at wider angles while the size and shape of the protein, including its associated waters of hydration, most strongly influence the low-to-mid-angle scattering. An envelope is built around the protein at a preselected distance sufficient to ensure the bulk character of the outermost solvent molecules. This distance was optimized by Park *et al.* (2009 ▸) where they examined values in the range 3–12 Å and found that 7 Å was large enough to contain all the non-bulk water for myoglobin and lysozyme, and they suggest this value is likely to be adequate for most proteins. Fitting data to the computed intensities uses only the scaling factor between the experimental and computed intensities and an optional constant subtraction. Of course, the values of a variety of parameters are key to the complex calculations of the MD trajectory and of the resulting scattering intensities, such as the water model, the force-field for the protein, the force constant applied to backbone atoms, the distance of the envelope to the protein surface *etc*. The influence of these parameters on the results was systematically investigated during the development of the *WAXSiS* program and default values chosen as proposed on the application website where protein atoms are placed in the AMBER03 force field and the solvent is described by the TIP3P water model. Certain parameters can be modified by the user before computing scattering intensities, but no value is subsequently adjusted to improve the fit to experimental data.

## 2. Methods

### 2.1. Sample details, data acquisition and reduction

Details of the samples measured, and original data collection and reduction are reported in the round-robin study [Tables 1 and S3 in Trewhella *et al.* (2022 ▸)] in accord with the publication guidelines of Trewhella *et al.* (2017 ▸). Provided here are the essential details required to understand the calculation of the consensus profiles, the fits using each modelling method and any additional information relating to the interpretation of the fits.

The pI values for each protein were calculated using *ProtParam* in *Expasy* (https://web.expasy.org/cgi-bin/protparam/protparam) with sequences from *UniProt*, identifiers (residue ranges): P24300 for xylose isomerase (pI 4.96), Q00511 (2–302) for urate oxidase (pI 7.16), F8W669 (1–190) for xylanase (pI 8.14), P00698 (19–147) for lysozyme (pI 9.32) and P61823 (27–150) for RNase A (pI 8.64)

### 2.2. Scattering data analysis

Scattering profiles are presented as *I*(*q*) versus *q*, where *q* is the amplitude of the momentum transfer between the incident and scattered waves expressed as (4πsinθ)/λ (θ is half the scattering angle and λ is the wavelength of the radiation). The *p*(*r*) function is the distribution of distances (*r*) between scattering centres within a scattering particle, weighted by the product of their scattering lengths, and is related to *I*(*q*) by Fourier transform. The *p*(*r*) profiles presented here were calculated by indirect Fourier transform using the programs *GNOM* (Svergun, 1992 ▸) [v5, as implemented in *PRIMUS/Qt* (v6.2.4) of the *ATSAS* suite (v3.2.1) (Manalastas-Cantos *et al.*, 2021 ▸)] or *BayesApp* (v1.3) (Hansen, 2000 ▸, 2012 ▸; Vestergaard & Hansen, 2006 ▸). Both *GNOM* and *BayesApp* automatically provide optimized solutions with estimated *d*_max_ values without requiring the user to specify *d*_max_. In the case of *GNOM*, these are provided as the result of an initial *AUTOGNOM* calculation that optimizes a total quality estimate parameter *Q*, after which the user can change *d*_max_. *Q* includes the χ^2^ value for the fit as well as perceptual criteria that are expressed mathematically, most importantly smoothness, stability and the absence of systematic deviations (Svergun, 1992 ▸). With *BayesApp*, the optimal solution is identified using Bayesian inference and there is an option to provide a first guess *d*_max_ value, which makes the algorithm more robust and faster. Unless otherwise specified, results reported are from *AUTOGNOM* or with *BayesApp* run with a first guess *d*_max_ that is free to change with optimization. *BayesApp* also assesses if errors are over or underestimated and provides error scaling adjustment factors (Larsen & Pedersen, 2021 ▸).

Guinier fits to experimental data were carried out using *AUTORG* in *PRIMUS/Qt* which finds the optimal linear fit region within a theoretical Guinier region to determine a ‘best’ *q*_min_ and *q*_max_. In some cases, *q*_max_ was adjusted to the theoretical limit for a homogeneous, globular scattering particle of *qR*_g_ ≃ 1.3 (Guinier & Fournet, 1955 ▸). Guinier fits to model profiles were done in *ORIGIN* (v2023b) by linear regression of plots of ln*I*(*q*) versus *q*^2^ up to *qR*_g_ ≃ 1.3 and *R*_g_ values were calculated from the slope of the fit (slope = −*R*_g_^2^/3). Plots for all figures were also generated using *ORIGIN* (v2023b).

Consensus data and custom *WAXSiS* calculations for each protein from the round-robin study were taken from the SASBDB depositions SASDPR4 (xylose isomerase), SASDPQ4 (urate oxidase), SASDPS4 (xylanase), SASDPT4 (lysozyme) and SASDPP4 (RNase A).

### 2.3. Scattering profile predictions

Predicted scattering profiles were modelled using the crystal structure coordinates deposited in the Protein Data Bank (PDB) with accession codes 1mnz for xylose isomerase (Nowak *et al.*, unpublished work), 3l8w for urate oxidase (Gabison *et al.*, 2010 ▸), 2dfc for xylanase (Watanabe *et al.*, 2006 ▸), 2vb1 for lysozyme (Wang *et al.*, 2007 ▸) and 7rsa for RNase A (Wlodawer *et al.*, 1988 ▸). For the xylose isomerase tetramer, a single N-terminal Met missing from the crystal structure was added to each chain in the coordinate file using *PyMOL* (The PyMOL Molecular Graphics System, Version 2.3.3 Schrödinger, LLC), which was also used to create Fig. 1 ▸. For the urate oxidase tetramer, the missing C-terminus (SLKSKL) from the 3l8w structure was added to each chain using *Modloop* (Fiser & Sali, 2003 ▸). For all proteins, modelled water and additional ions or ligands present in the coordinate files but not present in the solution conditions were removed.

Scattering profiles calculated using the crystal structure coordinates for each protein used the most recently available versions of each modelling program: *CRYSOL* (v3.2.1) as part of the *ATSAS* suite (v3.2.1) (https://www.embl-hamburg.de/biosaxs/software.html; Manalastas-Cantos *et al.*, 2021 ▸); *Pepsi-SAXS* Linux v3.0 (available at https://team.inria.fr/nano-d/software/pepsi-saxs/); *FoXS* calculations were via the website (https://modbase.compbio.ucsf.edu/foxs/, part of the *IMP* software package v2.19.0); *WAXSiS* via the website (https://waxsis.uni-saarland.de/).

*CRYSOL* (with the classic directional hydration layer) and *Pepsi-SAXS* fits to data included optimization of δρ and *r*_0_ and a constant adjustment. *CRYSOL* fits specified *q*_max_ = 1.0 Å^−1^, 1001 points in the profile, 70 spherical harmonics, Fibonacci grid 18, a constant adjustment and solvent density 0.335 Å^−1^ to match the calculated value based on chemical composition. *Pepsi-SAXS* calculations specified *q*_max_ = 1.0 Å^−1^, 1001 points in the profile and a constant adjustment. *FoXS* calculations refined parameters *c*_1_ and *c*_2_. *FoXS* calculations specified *q*_max_ = 0.999 Å^−1^, 1000 points in the profile and a constant adjustment.

*WAXSiS* calculations submitted to the website were performed with default options, except the bulk solvent density was set to 0.335 Å^−1^ and the ‘thorough’ mode selected where the MD simulations run longer times for improved convergence. In addition to the *WAXSiS* calculations via the website, custom *WAXSiS* calculations were available for each protein from the original round-robin study. The complete MD simulation systems for these custom *WAXSiS* calculations are available at Zenodo (https://zenodo.org/records/7057567) and a full description is provided in section S1 of the supporting information of Trewhella *et al.* (2022 ▸). Briefly, as for the calculations from the website, a spatial envelope was built around the protein at a distance of at least 7 Å from all solute atoms in all simulation frames with solvent atoms inside the envelope contributing to the SAS calculations to account for the modified density of the water molecules of hydration. The custom *WAXSiS* simulations were distinct in two main respects. First, they were performed using the *GROMACS* software (Abraham *et al.*, 2015 ▸) (version 2021.3) and run for longer times (50 ns) compared with the version available on the *WAXSiS* website that uses *Yasara* software (Krieger & Vriend, 2015 ▸). Second, the custom calculations included sodium, chloride and magnesium ions in the systems to match the experimental conditions. Interactions of the protein and ions were described with the AMBER99SB-ILDN (Lindorff–Larsen *et al.*, 2010 ▸; Hornak *et al.*, 2006 ▸) force field and using ion parameters described by Joung & Cheatham (2008 ▸). The buffer subtraction was carried out using simulation frames from pure-buffer simulation boxes whose salt content closely matched the respective protein simulations. Explicit-solvent SAXS calculations (Chatzimagas & Hub, 2022*a* ▸; Knight & Hub, 2015 ▸) were performed using the rerun functionality of an in-house modification of *GROMACS* (version 2018.8), as also implemented in the website *WAXSiS*.

### 2.4. Pseudo-experimental data calculations and fits

Pseudo-experimental *I*(*q*) data were generated for the tetrameric xylose isomerase (XI_1_) and a mixture of XI_1_ with an arbitrary ‘dimer of tetramers’ (XI_2_). First, coordinates for XI_2_ were generated using *PyMOL* and the XI_1_ coordinates (PDB entry 1mnz, with the added N-terminal me­thio­nines as described above). Predicted profiles for XI_1_ and XI_2_ were then calculated using *CRYSOL*, *Pepsi-SAXS* and *FoXS*. Errors were added to the predicted profiles, using error profiles from an individual experimental dataset from the original round-robin study, and random noise was also added. Pseudo-experimental *I*(*q*) datasets for XI_1_ with increasing ‘contamination’ of up to 10% XI_2_ were obtained by simple averaging of the proportionately scaled XI_1_ and XI_2_ profiles with standard error propagation performed using the *Average* operation in *PRIMUS/Qt*. The resulting series of pseudo-experimental datasets was fitted to the profile calculated for the tetramer structure (*i.e.* pure XI_1_) using *CRYSOL*, *Pepsi-SAXS* and *FoXS*, respectively, as described in Section 2.3 for the consensus profiles. In each case, this fitting was done using pseudo-experimental data calculated from XI_1_ and XI_2_ profiles generated using the same program, with the same protocol for adding errors and noise.

### 2.5. Consensus scattering profile calculations using maximum likelihood

The *ML-SAScombine* tool developed here (by AHL as an open-source python program, available at https://github.com/andreashlarsen/ML-SAScombine) combines data using maximum likelihood. The *ML-SAScombine* program can be divided into four steps:

Step (1) *Determine the scale factors and constant adjustments of data for optimal alignment*. One dataset is chosen as the reference data, by default it is the first dataset in the user-provided set of input data, but any curve containing **q** and **I** values can be used. Uncertainties from the reference data are not used. The reference dataset (**q**_ref_, **I**_ref_) is then linearly interpolated onto the *q* values of the second dataset (*q*, *I*, σ), in the range covered by both datasets. We note the vector notation applied here is **I** = [*I*_1_, *I*_2_,…, *I_N_*]. The interpolated intensities of the reference dataset (**I**_ref_) are fitted to the second dataset using a linear function *a*·**I**_ref_ + *b*. The resulting χ^2^ value is
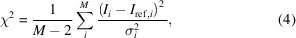
where *M* is the number of points in the second dataset that are also in the reference data. The degrees of freedom are *M* − 2 as two parameters are fitted. The resulting fit values (*a*, *b*) are used to align the second dataset to the reference data **I**_align_ = (**I** − *b*)/*a*, **σ**_align_ = **σ**/*a*. This procedure is repeated for all data.

Step (2) *Combine the data*. To combine the data, a *q* array is defined, either linearly or logarithmically. Then, each input datapoint (*q_i_*, *I_i_*, σ*_i_*) is assigned to a bin in the array (after alignment). Bins are annotated with the subscript *j*. The data are then combined using maximum likelihood (Taylor, 1997 ▸), with weights *w_i_* = *σ_i_*^−2^:
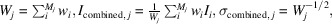
where the sums are over the *M_j_* datapoints assigned to the *j*th bin of the *q* array.

With this procedure, we note that the *q* values in the combined data are generally not equi-spaced as the final *q* values are also the weighted average of the points assigned to each bin:
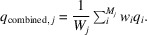
Though this does not generally give equispaced *q* values if the input data have different *q* values, it does give the most likely solution when combining data with non-identical *q* grids. Also, if no points are assigned to a given value in the predefined *q* array (*q_j_*), *i.e. M_j_* = 0, this bin is excluded from the combined dataset. Therefore, the number of points in the predefined *q* array specified by the user is a maximum number for the points in the combined data, and the number of points after removal of empty bins is generally slightly smaller.

Step (3) *Normalize the data*. The scaling is adjusted to obtain forward scattering of unity [*i.e.I*(0) = 1]. The default option is to divide the scattering profile *I*(*q*) by the average of the first five points, rendering the combined data unitless. Alternatively, an option is available that calls the program *AUTORG* (of the *ATSAS* suite) to normalize by division by *I*(0) from the Guinier fit. The constant background level is adjusted such that the datapoint with the lowest intensity has a value of 0.001 in the default mode, but an option is available for the user to input the constant background level, *e.g.* based on the dynamic range of a theoretical scattering profile they may have generated.

Step (4) *Ensure convergence of the alignment step*. Using (**q**_combined_, **I**_combined_) as reference data, steps (1) to (3) are repeated iteratively until the χ^2^ values [equation (4)] of the alignment step converge. This step ensures that the consensus data are independent of the reference dataset.

### 2.6. Revised protocol for calculating the updated consensus profiles

The previously published consensus profiles from the round-robin study were generated by first merging a single SEC-SAXS or low-concentration dataset with one or two of the higher concentration batch datasets from individual instruments. Outside the merge region, noisy SEC-SAXS data at higher *q* values and batch data affected by aggregation or interparticle interference at lower *q* values were discarded. The resulting merged scattering profile was evaluated according to the traditional criteria for consistency and quality. This procedure aimed to optimize the signal-to-noise over the broadest *q* range practically achievable. Independent datasets from different instruments were then combined using the program *DATCOMBINE* (https://www.embl-hamburg.de/biosaxs/manuals/datcombine.html) that optimizes the scaling and constant adjustments of the individual datasets using the Levenberg–Marquardt minimization (Moré *et al.*, 1984 ▸) of all pairwise χ^2^ comparisons among scattering profiles prepared on a common *q* grid. Errors are propagated simply as a plain average and filters can be used to remove (1) data with very large statistical errors that increase the plain average, and/or (2) data points that lie beyond the expected normal distribution of intensities at any given *q* value [using the modified *Z*-score from Iglewicz & Hoaglin (1993 ▸)].

With the *ML-SAScombine* tool, there is no need to exclude noisy data to optimize the errors. In addition, we decided on a strategy of combining all available SEC-SAXS and batch SAXS data without first merging data from the same instrument. As a result, significantly more data could be included in generating ‘updated’ consensus profiles compared with the ‘original’ consensus profiles from the round-robin study. Combining the larger amounts of data with *ML-SAScombine* was facilitated by work done in the round-robin study to evaluate the SEC-SAXS and low-concentration data with respect to monodispersity and interparticle interference, and to determine at what *q* values one could start including higher-concentration batch data without changing the shape of the resulting SAS profile. As a check, significant deviations of individual datasets from the consensus were identified using the plots of error-weighted residual differences and χ^2^ values for each individual dataset with respect to the combined result provided by *ML-SAScombine*. For these calculations, the experimental statistical errors are used but not those of the combined result. Typically, the *q*_min_ values for batch data inclusion were chosen to lie well beyond the Guinier region, before the first minimum in the scattering pattern and such that the Guinier and *p*(*r*) results were the same compared with combined pure SEC-SAXS data.

No outlier filter is incorporated in *ML-SAScombine*, although the user can choose to eliminate outliers when defining the input data. Indeed, this is important in the case of data with good statistics but poor-quality sample or instrumental conditions. Here, data points affected by parasitic scattering around the beam stop were excluded using a lower limit for a linear Guinier region (as determined by *AUTORG*).

## 3. Results

### 3.1. Evaluation of the updated consensus profiles and comparisons with the original set

With the above protocol, updated consensus profiles were readily obtained for four of the five proteins: xylose isomerase, urate oxidase, xylanase and RNase A (Table 1 ▸). However, initial attempts with the new protocol for lysozyme produced consensus data that gave a Guinier *R*_g_ value of 15.12 ± 0.02 Å, a value that is approximately half an Ångstrom larger than expected for lysozyme. Further, with a single exception, it was noted that all the SEC-SAXS data collected in the round robin gave Guinier *R*_g_ values in the range 14.9–15.5 Å. The lysozyme *R*_g_ values in the contributed datasets showed a greater variation than expected given the precision of the data and we had attributed this to a confounding combination of aggregation and interparticle interference effects (Trewhella *et al.*, 2022 ▸). This small, cysteine-rich protein whose structure is stabilized by several disulfide bonds has long been considered an undesirable standard for SAXS at synchrotron beamlines due to its high sensitivity to radiation damage that is enhanced with decreasing protein concentration. The SEC-SAXS data, predominantly from the highest-intensity sources and necessarily measured in the lower concentration ranges, appeared to be affected by very small amounts of radiation-induced aggregation despite the presence of free radical scavengers in the buffer. All the lysozyme batch data and SEC-SAXS data with Guinier *R*_g_ values >15 Å came from the brightest sources. Indeed, it had proven impossible to measure the lysozyme on the P12 BioSAXS instrument at Petra III without it precipitating and fouling the sample cell. Re-examination of the batch data yielded a set of twelve measurements from four instruments, including the sole laboratory based system and four of the less bright synchrotron instruments that gave Guinier *R*_g_ values of ∼14.5 Å. This set was selected to calculate the updated consensus profile.

For all five proteins, the mean χ^2^ values for individual datasets against their updated consensus (calculated as the cumulative χ^2^ divided by the total number of datasets, Table 1 ▸) were consistently in the range 0.84–1.09, with lysozyme and xylanase accounting for the smallest values as the contributing datasets were relatively fewer and all from quite low-concentration samples. For all the small proteins, the upper limit of the χ^2^ range was <1.2, while for the larger proteins, ∼10% of the included datasets gave χ^2^ values >1.2 (up to 1.37 for xylose isomerase and 1.65 for urate oxidase). However, checks of the residual difference plots calculated with respect to the consensus curve ensured that none of the included data showed systematic deviations >3σ. Also, additional checks were done by eliminating datasets with χ^2^ values in the higher range and affirming that no significant difference in the consensus was detected. The resulting updated consensus profiles for all five proteins are deposited in the SASBDB, along with the individual experimental datasets and the *ML-SAScombine* scripts used to generate them. These profiles were generated using a log *q*-binning that aids in resolving features in the higher-*q* regions.

Inspection of the original and updated consensus profiles after scaling and constant adjustment showed the same overall profile shape and plots of the errors [σ_expt_(*q*) versus *q*] showed smoother distributions for the updated consensus profiles, most notably for xylose isomerase (Fig. S1 of the supporting information). Further, there is a 25% reduction in average error magnitude for the updated consensus profiles for xylose isomerase in the *q* range 0.08–0.45 Å^−1^, a 66% reduction for urate oxidase in the *q* range 0.08–0.5 Å^−1^ and a 75% reduction for RNase A in the *q* range 0.05–0.675 Å^−1^. In each case these reductions are largely attributable to the greater number of batch datasets contributing to these regions. The average differences in error distributions for lysozyme and xylanase are less significant due to the more limited number of datasets suitable for combining. In the case of xylanase, dimer contamination in much of the data including, albeit small amounts, in most of the SEC-SAXS data was the limiting factor.

**Figure 2:**
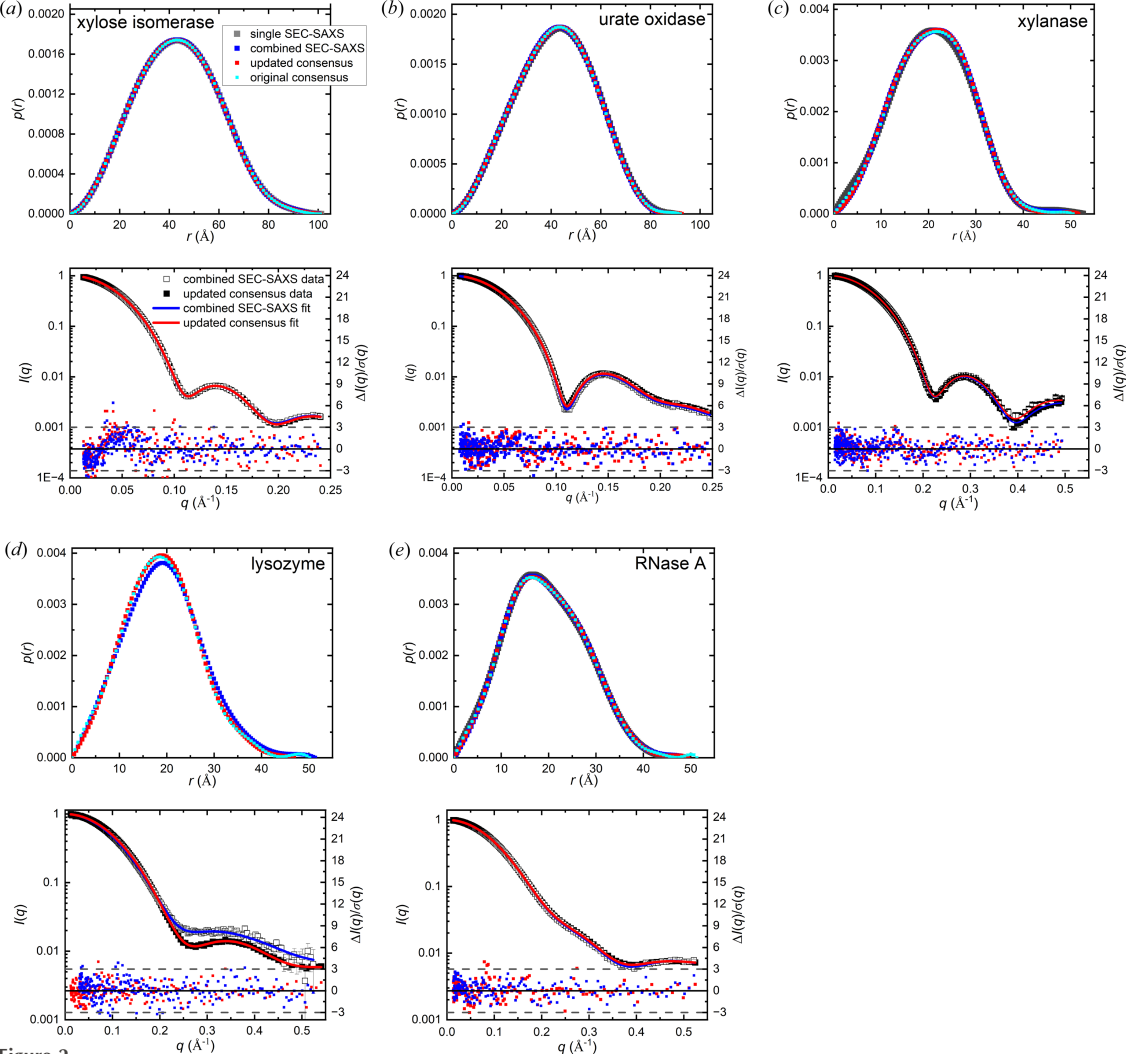
(*a*)–(*e*) Upper panels: overlaid *p*(*r*) plots for the updated and original consensus data, the combined SEC-SAXS data (using *ML-SAScombine*) and a single SEC-SAXS dataset (from the BioSAXS, Petra III EMBL-Hamburg, except for xylanase that is from the BioSAXS/WAXS, Australian Synchrotron). (*a*)–(*e*) Lower panels: *I*(*q*) versus *q* for the updated consensus and combined SEC-SAXS data overlayed with their *p*(*r*) fits (left axes) and the error-weighted residual difference plots below (right axes). Horizontal dashed lines indicate Δ*I*(*q*)/σ_expt_(*q*)^2^ = ±3. Error bars are mostly smaller than the symbols. The *p*(*r*) and *I*(*q*) plots are each normalized by *I*(0) division. The colour code in panels (*a*) are used for all, noting that there is no single SEC-SAXS *p*(*r*) profiles for lysozyme.

With the exception of lysozyme, the *p*(*r*) functions for each protein derived from the original and updated consensus profiles, the combined SEC-SAXS data and a single SEC-SAXS dataset are indistinguishable, and the corresponding derived structural parameters are the same within error (Tables 2 ▸ and 3 ▸; Fig. 2 ▸). For lysozyme, the *p*(*r*) functions from the original and updated consensus profiles are quite similar, yielding the same *p*(*r*)-derived *R*_g_ values and Guinier *R*_g_ values only marginally smaller for the updated consensus data (14.54 ± 0.01 Å) compared with the original (14.64 ± 0.05 Å). Notably, the Guinier region is linear to lower *qR*_g_ values for the updated consensus profile (0.17 compared with 0.22 for the original). However, the combined SEC-SAXS *p*(*r*) shows a small but significant right shift compared with the consensus that yields an increase in *R*_g_ of ∼0.5 Å, consistent with small amounts of aggregation present in the SEC-SAXS samples. Although the impact of beam brightness on the SEC-SAXS data for lysozyme was initially missed, it appears that its influence was minimal in the original consensus profile, likely due to the combined outlier and error filters used in *DATCOMBINE*.

The residual error-weighted difference plots for *p*(*r*) fits to the combined SEC-SAXS or updated consensus data show no significant systematic deviations and are predominantly within the expected ±3σ (Fig. 2 ▸, lower panels). Except for xylose isomerase, *p*(*r*) approached *d*_max_ with the expected smooth tangential approach to zero with no need to adjust *d*_max_ from that chosen by *AUTOGNOM*. In contrast, *AUTOGNOM* selected an unphysical *d*_max_ for xylose isomerase such that *p*(*r*) oscillated about zero for *r* values near *d*_max_, hence *d*_max_ was manually adjusted. Linear *q*-binning with a limit of 0.01 Å^−1^ produced essentially the same profile shape and the desired smooth tangential approach to zero with no need to adjust *d*_max_ from that chosen by *AUTOGNOM*. This kind of inconsistency with the *q*-binning was not observed for the other four proteins, each of which have significantly larger errors compared with xylose isomerase, which raises the question whether the small size of the errors for xylose isomerase reached a limit where the perceptual criteria used to determine the optimal transform in *GNOM* might be revisited.

In all cases, *p*(*r*) calculations using *BayesApp* gave very similar results to *GNOM*, and *BayesApp* assessments of the errors indicated they were, on average, underestimated by factors of 1.06–1.72 (Table 1 ▸). Re-gridding the input data of the original consensus calculation to a common *q* scale (using *DATCOMBINE* with no filters applied) facilitated calculation of the standard deviation from the mean *I*(*q*) at each *q* bin. Comparison of these standard deviations with the propagated errors in the original consensus profiles indicated that those errors were, on average, similarly underestimated by factors less than two: 1.72 for xylose isomerase, 1.10 for urate oxidase, 1.79 for xylanase, 1.58 for lysozyme and 1.09 for RNase A. Given the correction factors indicated by either method are all less than two, and different binning strategies lead to differences in the error distributions, no adjustment of errors has been made in the following.

### 3.2. Benchmarking with the updated consensus data

#### 3.2.1. Testing the robustness of model fits using different sets of combined data

**Figure 3:**
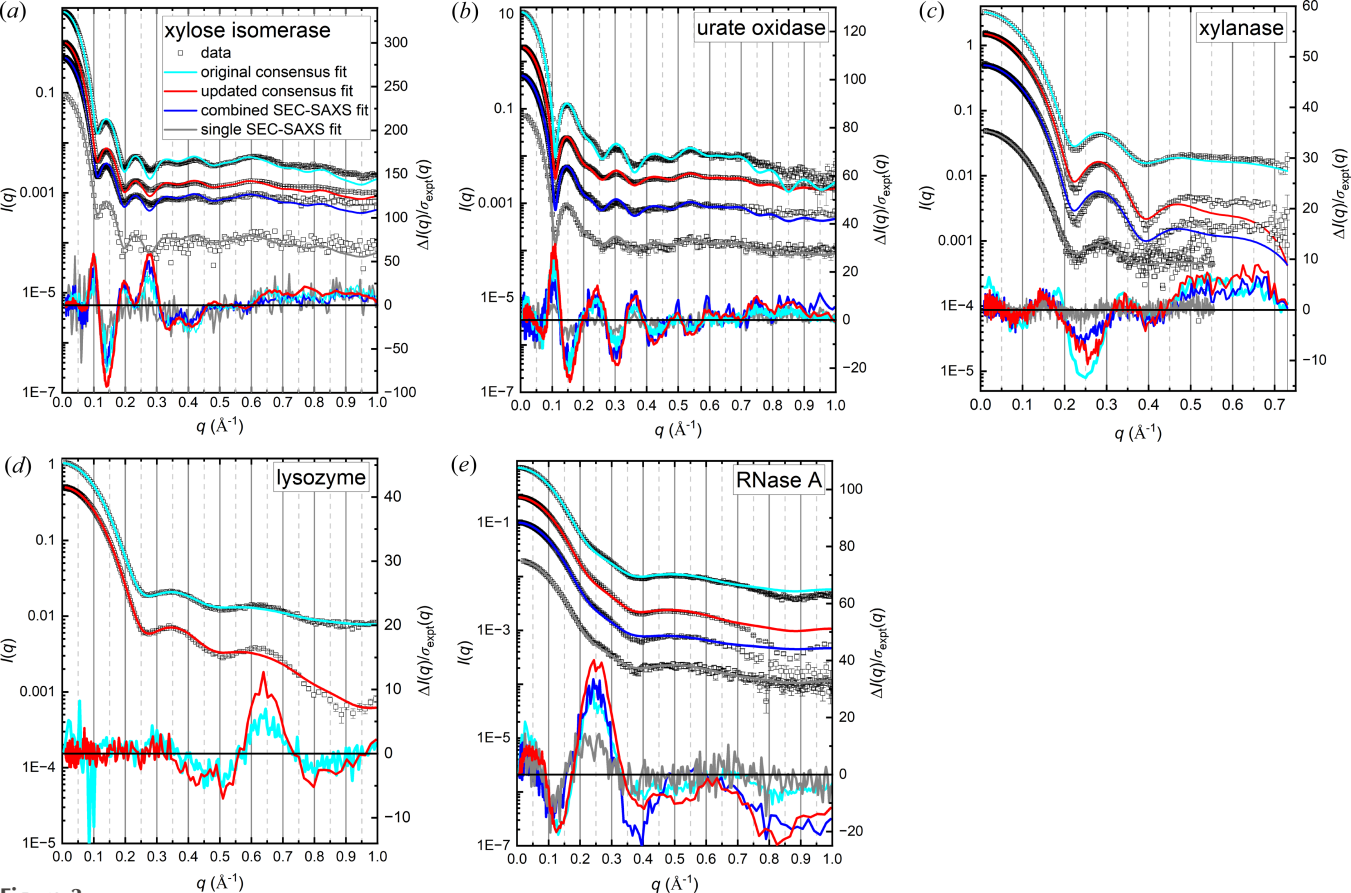
*CRYSOL* fits to *I*(*q*) versus *q* (left axes), offset for clarity, with error-weighted residual difference plots below (right axes) from the updated and original consensus profiles, the combined SEC-SAXS profiles from *ML-SAScombine* and a single SEC-SAXS measurement. Multiplication factors were applied to the differences for the combined SEC-SAXS data and single SEC-SAXS data (4 and 10, respectively) for xylose isomerase, (3 and 2) for urate oxidase and (3 and 4) for RNase A to better illustrate the similarities in the shapes of the difference plots. For xylose isomerase, a multiplication factor of 2 was applied to the original consensus data. Note: log binning is used for the updated consensus and combined SEC-SAXS data, which has the effect of reducing the noise especially at high-*q* values, while the original consensus data are binned linearly. The colour code in (*a*) is used for all panels.

Beyond comparing the derived structural parameters for different combined datasets, the robustness of residual difference plots for model fitting was first demonstrated by comparing *CRYSOL* fits for each protein with the original and updated consensus profiles, and (except for lysozyme) the results for combined pure SEC-SAXS data and a single SEC-SAXS measurement (Fig. 3 ▸). The choice of any of the modelling programs for this comparison is arbitrary as the results are equally well demonstrated with all of them. For each protein, *CRYSOL* fits to the set of *I*(*q*) profiles show the same shapes in the error-weighted residual difference plots with only the relative noise and magnitude of features changing due to differences in the relative magnitude of the propagated experimental statistical errors [σ_expt_^2^(*q*)]. For xylose isomerase, this difference is greatest due to the inclusion of more data from higher concentration samples in the updated consensus profiles compared with the other proteins. For the similar-sized urate oxidase, data were collected in a narrow protein concentration range with an upper limit of ∼5 mg ml^−1^. In summary, for each protein the overall shape of the error-weighted residual difference plot was neither affected by the different approaches to combining data, nor did it change when the different sets of data were combined.

#### 3.2.2. Assessing fits to the consensus data obtained with models with an implicit hydration layer

**Figure 4:**
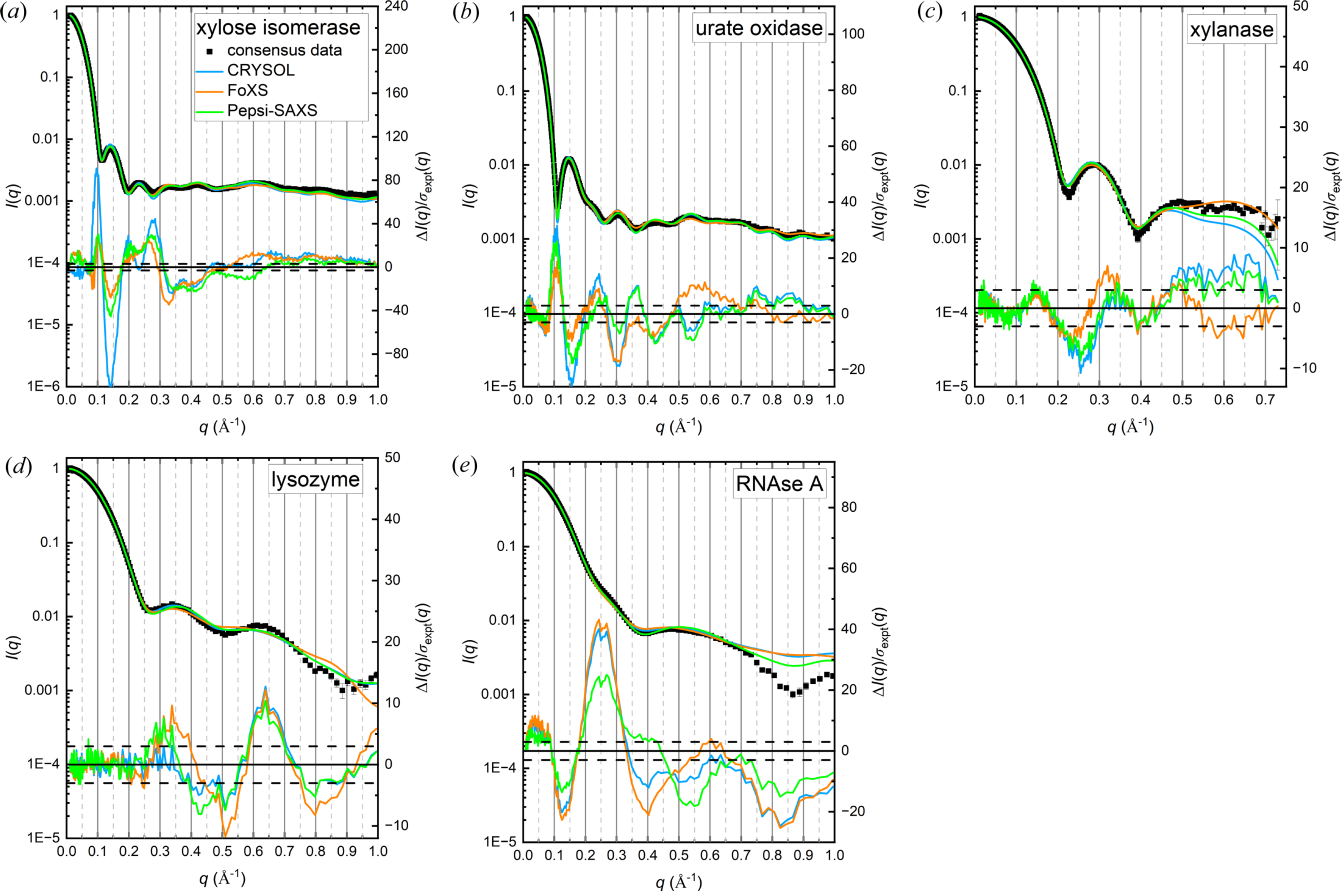
Updated consensus *I*(*q*) versus *q* [log *q*-binning to aid in resolving features in the higher-*q* regime (*q* > 0.5 Å^−1^)] with the fitted model profiles (left axes) and corresponding error-weighted residual difference plots below (right axes) for models with an implicit hydration layer. The horizontal dashed lines indicate Δ*I*(*q*)/σ_expt_(*q*)^2^ = ±3. The colour code in (*a*) is used for all panels.

The updated consensus profiles for each protein, as described in Table 1 ▸, were fit using the three modelling programs with an implicit hydrogen layer: *CRYSOL* (with the classic directional hydration layer as well as with dummy waters), *FoXS* and *Pepsi-SAXS*. The optimized adjustable parameters, constant adjustment and χ^2^ values for each fit are compared in Table 4 ▸. The σ_expt_(*q*)-weighted residual difference plots reveal significant differences between the model and experiment in all cases (Fig. 4 ▸). *CRYSOL* calculations run with the dummy waters model yields essentially the same results as for the directional hydration layer, except in the case of urate oxidase where χ^2^ is reduced and there is a corresponding reduction in the magnitude of features in the residual difference plot, with no difference in overall shape for *q* < 0.5 Å^−1^, but some variations for *q* > 0.5 Å^−1^ (Fig. S2).

Except for RNase A, the residual difference profiles display oscillations in approximate register with the well defined subsidiary maxima and minima in the *I*(*q*) profiles that reflect the overall size and shape of the scattering particle (protein plus hydration layer). For xylose isomerase and urate oxidase, *FoXS* diverges from this pattern around 0.5 Å^−1^. The RNase A profile is distinct from the others in that the characteristic subsidiary maxima for a globular scatterer are not well resolved and the largest feature in the residual difference plots is a broad peak in the mid-*q* region that can be dominated by scattering from pairs of atoms between domains. There is evidence for subtle domain motions in RNaseA (Vitagliano *et al.*, 2002 ▸) that could account for these features.

Some general observations from inspection of the parameters in Table 4 ▸ include the following:

(i) The χ^2^ values are all large and of similar order of magnitude across the three programs for a given protein. The largest differences are in the χ^2^ values for fits to xylose isomerase that has the smallest errors; *CRYSOL* values are as much as four times those for *FoXS* and *Pepsi-SAXS*. Uniquely, the *CRYSOL* fit for urate oxidase with dummy waters selected for the hydration model yields a χ^2^ value that is about half that for the directional hydration layer model and much closer to the values obtained with *Pepsi-SAXS* and *FoXS*.

(ii) Inspection of the fit parameters for a given protein using the different programs reveals inconsistencies. For example, the atomic radii adjustments (*r*_Sc_ or *c*_1_) for xylanase in all three programs are ∼1.02, but the percentage change in contrast for the hydration layer for *CRYSOL* (%δρ) (directional hydration layer) is less than half the value for *Pepsi-SAXS*, whereas *FoXS* assigns a negative *c*_2_ indicating a solvent layer with a negative contrast. *Pepsi-SAXS* and *FoXS* calculations consistently optimize with atomic radii adjustments in the range 1.01–1.03, whereas for *CRYSOL* the values are in the range 1.017–1.094 with the largest values (1.068–1.094) for the bigger proteins plus the highly charged lysozyme. For *Pepsi-SAXS* only, there is an evident steady increase in the hydration layer contrast with decreasing protein size, noting that *Pepsi-SAXS* assumes the water layer thickness is 3 Å for RNase A and lysozyme (each <15 Å *R*_g_), 5 Å for urate oxidase and xylose isomerase (each >20 Å *R*_g_), and an intermediate value of ∼3.4 Å for xylanase (*R*_g_ ∼16 Å).

(iii) *FoXS* optimizes to increasingly negative values of *c*_2_ in fitting the consensus profiles for xylose isomerase (*c*_2_ = −0.19), lysozyme (*c*_2_ = −0.49) and xylanase (*c*_2_ = −0.84), implying the hydration layer electron density is increasingly less than that of the bulk solvent for these proteins. Among these, xylanase carries the smallest net charge (pI 8.64 in a solution of pH 7.5), xylose isomerase carries a somewhat larger net charge (pI 4.96 in a solution of pH 7.5), whereas lysozyme carries by far the largest net charge among all the proteins (pI 9.32 in a solution of pH 4.5). These observations are at odds with the idea that the density of the hydration layer can be on average lower than that of bulk water, depending on the amount of surface charge.

(iv) *CRYSOL*, *Pepsi-SAXS* and *FoXS* all optimize their fits to the RNase A profile with the largest value for the hydration layer contrast among the five proteins, adding weight to the idea that for RNase A in solution there is a dynamic conformational ensemble for which the average conformation is somewhat larger than the crystal structure coordinates predict.

In light of the above, it is instructive to look at the trends in adjustable parameters (Table 5 ▸) for implicit hydration layers when modelling the pseudo-experimental data generated for the xylose isomerase tetramer (XI_1_) with increasing amounts of contamination of an arbitrary dimer of tetramers (XI_2_) (see Section 2.4). These data were fitted to the predicted profile for the pure tetramer using *CRYSOL*, *Pepsi-SAXS* and *FoXS*. With increasing XI_2_, the total excluded volume and hydration layer contrast are variously adjusted to minimize what is a real structural difference. With increasing XI_2_, *CRYSOL* increases %δρ to as much as 21%, while *r*_Sc_ values are in the range 0.98–1.0. The restriction on δρ_max_ in *Pepsi-SAXS* to 0.0334 e Å^−3^ means the optimal δρ is always the maximum allowed value, while *r*_Sc_ steadily increases to 1.03. For *FoXS*, *c*_1_ is in the range 1.0–1.03 while the contrast of the hydration layer steadily increases via *c*_2_ with increasing XI_2_. Curiously, *FoXS* applies a negative *c*_2_ value in fitting its own pure monomer profile.

#### 3.2.3. Models with explicit solvent using molecular dynamics

**Figure 5:**
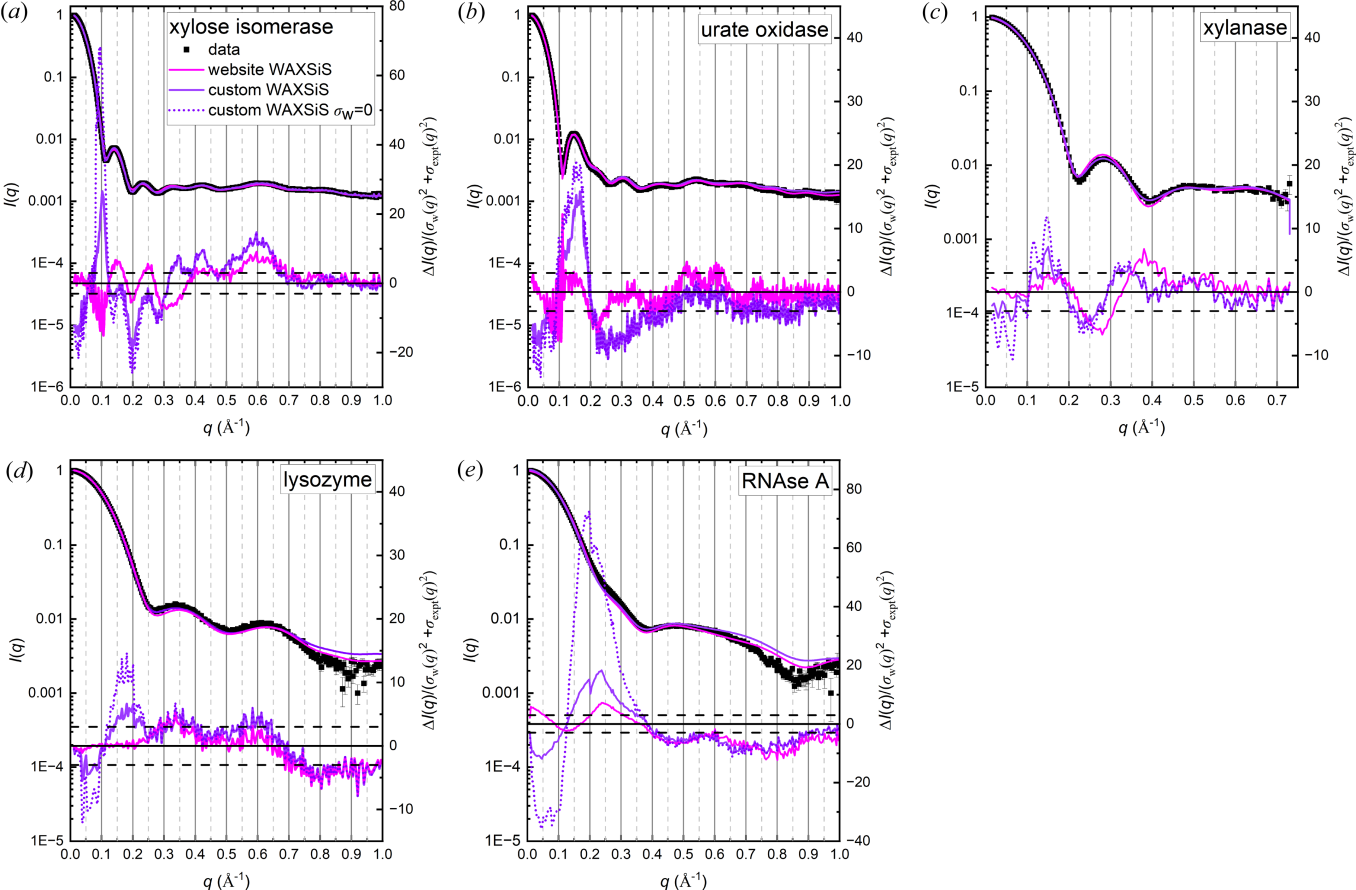
Updated consensus *I*(*q*) versus *q* (linear *q*-binning, see Section 3.2.3) with the fitted model profiles (left axes) and the corresponding error-weighted residual difference plots below (right axes) for website *WAXSiS* and custom *WAXSiS*. The horizontal dashed lines indicate Δ*I*(*q*)/[σ_expt_(*q*)^2^ + σ_w_(*q*)^2^]^1/2^ = ±3. The colour code in (*a*) is used for all panels.

The predicted scattering profile for each protein was calculated by submission of the respective crystal structure coordinates to the *WAXSiS* website using the thorough mode to improve convergence (hereafter referred to as the ‘website *WAXSiS*’ calculation). The error-weighted residual differences with the updated consensus profiles were then compared for the custom *WAXSiS* calculations round-robin study (hereafter referred to as the ‘custom *WAXSiS*’ calculation) versus the website *WAXSiS* (Fig. 5 ▸, Table 6 ▸). For this comparison, it was necessary to first produce consensus profiles compatible with the *q* grid of the custom *WAXSiS* profile deposited with the *SASBDB*, which is linear with constant Δ*q* = 0.002 Å^−1^. This was done by specifying a linear *q* grid in *ML-SAScombine* (1000 linear bins, except for lysozyme and RNase A where it was 251 bins, for *q* = 0–1 Å) and then re-gridding the resulting consensus profile to a common grid with the custom *WAXSiS* profile. The different *q* grids result in slightly different error distributions, and therefore the *CRYSOL*, *Pepsi-SAXS* and *FoXS* model calculations were repeated with the custom *WAXSiS* compatible consensus profiles to facilitate direct comparisons. While there are small variations in the relative magnitudes of features in the low-to-mid *q* regime (compare Figs. 4 ▸ and 5 ▸ with Fig. S3) and some variation in the adjustable parameters (Table 4 ▸ compared with Table S1 of the supporting information), the results are substantially the same.

Comparison of the website *WAXSiS* and custom *WAXSiS* fits with those using the implicit hydration layer are additionally complicated by the fact that the *WAXSiS* model profiles include statistical errors [σ_w_(*q*)] generated when predicted SAXS profiles are averaged over multiple simulation frames due to small fluctuations in the contribution of hydration water molecules to the scattering. Like the scattering profile itself, the σ_w_(*q*) profile decreases rapidly from a maximum at zero with increasing *q* (Fig. S4). The residual differences for the calculated versus experimental profile are calculated with the weighting [σ_w_(*q*) ^2^ + σ_expt_(*q*)^2^]^1/2^. Depending on the relative magnitudes of σ_w_(*q*) and σ_expt_(*q*), there can be a significant influence on χ^2^ and the associated residual difference plot. For a single SAXS measurement, it has generally been the case that σ_w_(*q*) was much smaller than σ_expt_(*q*); however, this is not the case for the consensus data, especially in the low-to-mid *q* region (Fig. S4). Indeed, for the website *WAXSiS* calculation (simulation times in the hundreds of picoseconds with 259–1341 frames averaged, Table 6 ▸) the *q* values where the ratio σ_w_(*q*)/σ_expt_(*q*) becomes smaller than 1.0 is between 0.3 and 0.7 Å^−1^. The magnitudes of σ_w_(*q*) values decrease as the simulation is run for longer times and more frames are averaged. It is also the case that, as the size of the protein decreases, the ratio of surface area to volume increases and hence also the relative contribution to the scattering of the hydration water molecules and of the surface side-chain atomic fluctuations. As a result, the simulations will take longer to converge. The custom *WAXSiS* calculations from the round-robin study were run for 50 ns, writing frames every 10 ps (for 5000 frames) to achieve greater convergence and resulted in significantly smaller statistical errors compared with those from the web submission (Fig. S4). To facilitate direct comparison of equivalent χ^2^ values with those calculated for models with an implicit hydration layer, a complete set was calculated for the custom and website *WAXSiS* profiles with *σ*_w_(*q*) set to zero (Table S2). Even with the moderating influence of the *WAXSiS* errors removed, with the notable exception of RNase A, the χ^2^ values compare favourably with those using an implicit hydration layer.

The custom *WAXSiS* with its generally smaller errors yielded higher χ^2^ values compared with the website *WAXSiS* by factors of 2–5 except for xylanase, which shows no significant difference (Table 6 ▸). The features in the corresponding residual differences are accordingly larger, notably for *q* ≲ 0.4 Å^−1^ (Fig. 5 ▸). For each protein, the residual difference plots for the custom and website *WAXSiS* calculations eventually converge with increasing *q*. To assess the influence of the *WAXSiS* statistical errors on the residual differences for the custom *WAXSiS* profile, the result for σ_w_(*q*) = 0 is superposed on the plots in Fig. 5 ▸ (dotted lines). The same features are resolved, just with increased magnitude in the region where σ_w_(*q*)/σ_expt_(*q*) is > 1, and the plots converge at *q* ≃ 0.2 Å^−1^ except for RNase A where the convergence is closer to *q* ≃ 0.4 Å^−1^.

The custom *WAXSiS* residual difference plot for xylose isomerase exhibits features that appear to reflect the subsidiary maxima in the consensus profiles but are not in register with the implicit hydration layer models. For urate oxidase, the *WAXSiS* profiles have an entirely different shape. Xylanase and lysozyme residual differences for all model fits are at least an order of magnitude smaller than observed for the other proteins and in the case of xylanase insignificantly different between the custom and website calculations, while lysozyme shows an additional feature for *q* < 0.25 Å^−1^ for the custom *WAXSiS* that is also reflected in a somewhat higher χ^2^ value. Finally, RNase A stands out for the significantly large broad feature centred at *q* ≃ 0.2 Å^−1^ that is dramatically increased (by a factor of ∼4) for the custom *WAXSiS* profile when σ_w_(*q*) is not considered, highlighting the inadequacy of the crystal structure model and consistent with the idea that there are domain motions for the protein in solution that must be included for accurate simulation.

#### 3.2.4. *R*_g_ values from implicit versus explicit hydration layer models compared with the experiment

**Figure 6:**
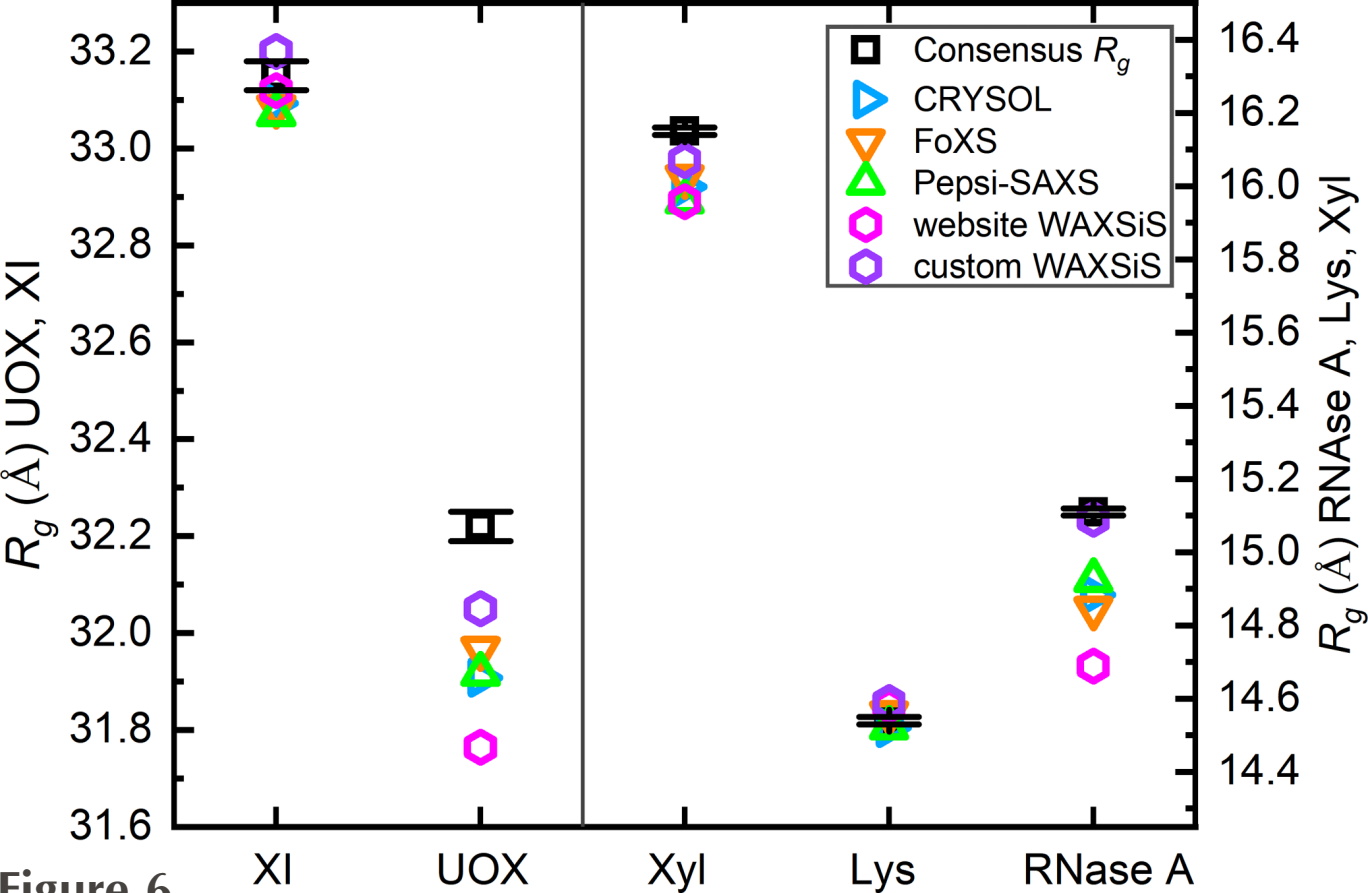
Guinier *R*_g_ values for xylose isomerase (XI) and urate oxidase (UOX) (left axis); and for xylanase (Xyl), lysozyme (Lys) and RNase A (right axis) from the updated consensus profile (with error bars) and those calculated from Guinier analysis of the fitted model profiles (*qR*_g_ < 1.3). Numerical *R*_g_ values are given in Table S2

The *R*_g_ values derived from Guinier analysis of the predicted profiles for each modelling approach were compared with values from the updated consensus (Fig. 6 ▸, see Table S2 for the values). All the predictive methods give *R*_g_ values within a few tenths of an Ångstrom of those calculated from the consensus profiles with some noteworthy differences. Unsurprisingly, the Guinier *R*_g_ values calculated from models with an implicit hydration layer cluster tightly as all three programs effectively optimize δρ and adjust the total excluded volume to fit the experiment and the fitting is dominated by the relatively high statistical precision, low-*q* data that determines the experimental *R*_g_. For xylose isomerase and lysozyme there is agreement within experimental error for all fitted models. For the other three proteins, the values from the various fits are less than the consensus experiment values. Those using an implicit hydration layer yielded *R*_g_ values that are lower than experiment by 0.13–0.36 Å, the website *WAXSiS*-derived values are even lower (by 0.19–0.46 Å), whereas the custom *WAXSiS*-derived values that are the closest to experiment with the largest deviation for urate oxidase (lower by 0.17 Å). Aside from longer simulation times, the custom *WAXSiS* calculations included sodium, chloride and magnesium ions in the systems to match the experimental conditions, which currently is not an option for the website *WAXSiS*.

## 4. Discussion and conclusions

The round-robin study was the first attempt to quantitatively assess the reproducibility of the solvent-subtracted scattering profile for a set of proteins in solution prepared, to the extent possible, under identical conditions. Evaluating the reproducibility and combining the SAXS data to obtain consensus scattering profiles with reliable error estimates for each protein that could be used for benchmarking predictive methods presented numerous challenges. The contributed data were collected with different instrument configurations that gave widely varying *q* ranges, *q* grids and *q* resolutions on beamlines, with orders of magnitude differences in brightness and hence large differences in statistical precision. At the time of the round-robin study, there was no tool available to combine such SAS data in a standard way to create a consensus profile. The solvent-subtraction uncertainty required a global optimization of constant adjustments to the set of data to be combined. Though statistical error estimates for the contributed data were validated by comparing all-pairwise solvent measurements for each dataset, there remained the challenge of minimizing errors while accurately propagating errors from the various contributed data.

The *DATCOMBINE* program was a first of its kind and was developed in response to a request for urgent help by the round-robin study data analysis team. Rapid development was facilitated by the availability of well tested subroutines within the *ATSAS* suite that could be combined to provide a tool. As a result, errors were propagated as the plain average with the consequential requirement to remove the noisiest data to minimize the errors in the combined dataset. With the new *ML-SAScombine* program, the use of maximum likelihood meant there was no need to filter the noisiest data to optimize the errors. Further, the revised protocol for combining data allowed for significantly more datasets to be included, resulting in smaller average magnitudes and greater smoothness in error distributions for xylose isomerase, urate oxidase and RNase A. For lysozyme and xylanase there was no significant improvement due to the combination of their small size and the limitations on the number of available datasets that were free of artefacts.

Our new protocol for combining data brought to light the fact that most of the SEC-SAXS data for lysozyme had a small degree of aggregation that was not as evident with individual measurements compared with the combined SEC-SAXS data. A recent study of different modes of SEC-SAXS data acquisition at the BioSAXS Beamline 4–2 of the Stanford Synchrotron Radiation Lightsource (SSRL) for bovine serum albumin (BSA) and lysozyme reported results for measurements with different photon energy (11 versus 13 keV), with and without 5 m*M* di­thio­threitol (DTT), different cell cleaning protocols, different sample configurations (*e.g.* co-flow versus standard flow) (Matsui *et al.*, 2024 ▸). The reported *R*_g_ values for lysozyme (in 50 m*M* sodium acetate pH 4.8, 150 m*M* NaCl) with the various combinations of conditions tested were in the range 14.36–14.66 Å, which compares well with the value of 14.54 ± 0.01 Å from the updated consensus profile from measurements in 50 m*M* sodium citrate, pH 4.5, 150 m*M* NaCl. The authors conclude that radiation damage and sample cell fouling for SEC-SAXS measurements generally, and for lysozyme specifically, can be mitigated by the presence of a free-radical scavenger (they recommend 3–5 m*M* DTT), a co-flow sample configuration, additional sample cell cleaning and using a higher photon energy where practical (they measured at 13 and 11 keV). The latter is of interest with respect to the P12 BioSAXS instrument (Petra III) where the photon energy is 10 keV and lysozyme measurements are impossible. The SSRL study perhaps provides a template for better standardizing the conditions for SEC-SAXS measurement for a future round robin with lysozyme and potentially BSA.

Although all the modelling methods predict the general features of the consensus profiles for each protein, the error-weighted residual difference plots showed well defined features that extend to 30–270 times σ_expt_(*q*) in the low-*q* regions. Smaller differences are observed out to *q* = 1.0 Å, where thermal fluctuations in the protein and solvent will dominate. The *p*(*r*) fits to the consensus data all show deviations largely within the expected ±3σ, which gives weight to the conclusions that the observed features reflect real differences between the models and experiment. With the exception of the *WAXSiS* fit to urate oxidase, the residual differences in the low-to-mid *q*-regime (<0.5 Å^−1^) largely reflect the structure of the subsidiary maxima in the profiles, strongly suggesting small but significant differences between the experiment and model in the apparent size of the protein that includes the contribution of the water molecules of hydration. In each case, *WAXSiS* shows a different pattern, with similar oscillations in the case of xylose isomerase, but not in register with the other methods, while for urate oxidase the pattern is markedly different and not reflective of the subsidiary maxima in *I*(*q*). Notably, urate oxidase is unique among the proteins in that it has a central water channel and for this protein only, the *CRYSOL* calculation with dummy waters provided an improved fit compared with the directional hydration shell that, as assessed by χ^2^, was more comparable to that obtained from *FoXS* and *Pepsi-SAXS*.

For RNase A, the large-amplitude broad peak in the mid-*q* region of the residual difference plots is consistent with our previous interpretation that there are domain motions in RNase A (Trewhella *et al.*, 2022 ▸). This interpretation is supported by NMR experiments that show subtle conformational differences in the NMR ensemble for RNase A in solution in the absence of active site-bound ligand (Santoro *et al.*, 1993 ▸). Crystallographic studies have further quantified domain motions in RNase A that are the result of a small-hinge bending motion between two β-sheets that form a V-shaped motif with the hinge defined by pairs of residues at the base of the V and connecting the three strands forming its sides (Vitagliano *et al.*, 2002 ▸) (see Fig. 1 ▸). Competitive inhibitor binding at the enzyme active site results in a small reduction in the hinge angle (from 92.8 to 90.9°), while there is an increase (from 90.9 to 93.1°) on inhibitor release. These structural transitions are reversible in the crystalline state and are consistent with the observations of dynamical behaviour in RNase A that is required for catalytic function (Rasmussen *et al.*, 1992 ▸). The residual difference plot for the custom *WAXSiS* fit to RNase A when the modulating impact of the *WAXSiS* generated statistical errors, σ_w_(*q*), are not considered is dramatically worse than any of the other fits. Further, each method with the implicit hydration layer optimizes to a relatively high contrast for the hydration layer that would effectively make the protein appear larger. For this example, where the evidence is that the solution structure is not well represented by a single average structure, we see how adjustable parameters can artificially improve the fit of experimental data and effectively mask differences in the solution scattering profile due to genuine conformational differences between crystal and solution. These results for RNase A, in combination with results obtained when modelling the pseudo-experimental data for the xylose isomerase tetramer with an increasing proportion of the dimer of tetramers, demonstrate the power of adjustable parameters in model fitting and their potential for arbitrariness.

Without the benefit of adjustable parameters, the all-atom explicit solvent molecular dynamics *WAXSiS* calculations predict the consensus profiles at least equally well, and in some cases better than any of the models using an implicit hydration layer and two adjustable parameters, except for RNase A where there is a real structural difference. Further, the improved agreement with experiment for *R*_g_ values from custom versus website *WAXSiS* calculations suggest the inclusion of ions in the solvent to match the experiment is a significant improvement in the MD model. Indeed, the presence of ions in the solvent, already studied by the Hub group several years ago (Ivanović *et al.*, 2018 ▸) might be expected to result in some transient binding to charged groups on the protein surface and may also affect the distribution of hydration water molecules during the simulation. Furthermore, in the case of a significantly charged protein, the immediate surroundings of the protein surface will be enriched in counterions that contribute to scattering while their concentration decreases with increasing distance from the surface down to that of the bulk according to the Debye–Hückel formalism (Ivanović *et al.*, 2018 ▸). The small increases in *R*_g_ values with the custom *WAXSiS* compared with the website *WAXSiS* suggests that the higher salt concentration in solution for this highly charged protein might at least in part account for the slightly larger *R*_g_ value from the updated consensus profile 14.54 ± 0.01 Å in 150 m*M* NaCl compared with *R*_g_ = 14.3 ± 0.04 Å in 50 m*M* NaCl from data recorded on the X33 EMBL beamline (DESY, Hamburg) (Mylonas & Svergun, 2007 ▸). These observations herald the exciting prospect of using the updated consensus profiles to test and further develop explicit all-atom solute and solvent MD simulations that can be rigorously tested against high-quality benchmarking data.

For *CRYSOL*, *Pepsi-SAXS* and *FoXS*, a significant driver in the development of these programs has been to increase the speed of the calculations. With automation and high-brilliance X-ray beamlines, there has been a rapidly growing interest in screening applications with SAXS, including for studying biomolecular interactions (*e.g.* Chen *et al.*, 2018 ▸) and drug development [for recent reviews see the literature (Trewhella, 2022 ▸; Brosey & Tainer, 2019 ▸)]. The computational resources and times required today to perform MD calculations are prohibitive for these kinds of applications; the website *WAXSiS* calculations in the thorough mode took 10–20 min in real time. On the other hand, if the questions being asked are about understanding in detail the influence of solvation and dynamic fluctuations on the scattering profile, or for testing our understanding of the basic physics and chemistry of these complex systems (*e.g.* the influence of force fields, ion parameters, precise solvent conditions, the structure in the water molecules of hydration, ion binding) the MD approach is essential.

In the final analysis, methods invoking an implicit hydration layer have the advantage of speed and, used with care to understand the influence of adjustable parameters, are useful for rapidly assessing the quality of the fit of a solution SAS profile to a model. They are most powerful for detecting changes in the association states of components in solution or sufficiently large conformational differences with structural models. All-atom solute and solvent molecular dynamics simulation take longer but are less susceptible to false positives. Further, as they are derived from the known physics and chemistry of the system, they can account for thermal fluctuations in atomic positions and more accurately represent the solution conditions, including the water molecules of hydration.

## Supplementary Material

SASBDB reference: ribonuclease A, SASDUA4

SASBDB reference: urate oxidase, SASDUB4

SASBDB reference: xylose isomerase, SASDUC4

SASBDB reference: xylanase, SASDUD4

SASBDB reference: lysozyme, SASDUE4

Supporting figures and tables. DOI: 10.1107/S205225252400486X/be5296sup1.pdf

## Figures and Tables

**Table 1:** Datasets included in the *ML-SAScombine* calculations for updated consensus data that were used for Guinier and *p*(*r*) analyses (Fig. 2 ▸, Tables 2 ▸ and 3 ▸) *N*_SS_, *N*_batch_, *N*_I__(*q*)_, *N*_inst_, *N*_bin_, are the numbers of SEC-SAXS profiles, batch profiles, total *I*(*q*) profiles combined, contributing instruments, bins specified for *ML-SAScombine*. For RNase A and urate oxidase, the *q*_min_ values for the batch data were spread over the ranges indicated in square brackets to minimize effects of the changes in error magnitude around the merge region.

Protein	*N*_SS_	SEC-SAXS *q*_min_ (Å^−1^)	*N*_batch_	Batch *q*_min_ (Å^−1^)	*N*_I__(*q*)_	*N*_inst_	*N*_bin_	Cumulative χ^2^ value†/*N*_I__(*q*)_ (maximum χ^2^)	Error adjustment factor per *BayesApp*
Xylose isomerase	6	0.01	4	0.01	29	10	501	1.04 (1.37)	1.72
			16	0.08					
			3	0.3					
Urate oxidase	9	0.01	25	[0.00788, 0.00812]	34	9	501	1.09(1.65)	1.28
Xylanase	4	0.01	11	0.18	17	7	501	0.84 (1.13)	1.06
			2	0.25					
Lysozyme	0	–	11	0.01	12	5	251	0.92 (1.15)	1.14
			1	0.5					
RNase A	6	0.005	12	[0.075, 0.085]	24	9	251	0.96 (1.16)	1.29
			6	[0.10, 0.20]					

† The cumulative χ^2^ value = Σχ^2^ for each contributing experimental *I*(*q*) calculated according to equation (4).

**Table 2:** Comparison of Guinier parameters derived from the original and updated consensus profiles *R*_g_ values are from *AUTORG* with *qR*_g_ ≤ 1.3.

	Original consensus		Updated consensus		*ML-SAScombine* SEC-SAXS data only	
	*R*_g_ (Å)	*qR*_g_ range (Å^−1^)	*R*_g_ (Å)	*qR*_g_ range (Å^−1^)	*R*_g_ (Å)	*qR*_g_ range (Å^−1^)
Xylose isomerase	33.15 ± 0.04	0.27–1.29	33.15 ± 0.03	0.42–1.29	33.17 ± 0.03	0.42–1.30
Urate oxidase	32.30 ± 0.06	0.26–1.29	32.22 ± 0.03	0.40–1.30	32.21 ± 0.03	0.31–1.30
Xylanase	16.12 ± 0.02	0.24–1.29	16.15 ± 0.01	0.24–1.29	16.15 ± 0.01	0.22–1.30
Lysozyme	14.64 ± 0.05	0.22–1.24	14.54 ± 0.01	0.17–1.30	15.08 ± 0.02	0.45–1.29
RNase A	15.13 ± 0.02	0.15–1.29	15.11 ± 0.01	0.20–1.28	15.14 ± 0.01	0.20–1.29

**Table 3:** Comparison of *p*(*r*)-derived parameters for the original and updated consensus profiles Values for the original consensus data are taken from Trewhella *et al.* (2022 ▸). The *GNOM* total quality estimate *Q* is unitless with the range 0–1, where *Q* ≥ 0.9 is considered an excellent solution, 0.9 > *Q* ≥ 0.8 is very good, 0.8 > *Q* ≥ 0.7 is good, and 0.7 > *Q* ≥ 0.6 is reasonable. The relatively low *Q* value for xylose isomerase is associated with very high χ^2^ values due to the very small statistical uncertainties for these data.

	Original consensus data			Updated consensus data			*ML-SAScombine* SEC-SAXS data only		
	*R*_g_ (Å)	*d*_max_ (Å)	*Q*	*R*_g_ (Å)	*d*_max_ (Å)	*Q*	*R*_g_ (Å)	*d*_max_ (Å)	*Q*
Xylose isomerase	32.93 ± 0.01	101.0	0.67	32.89 ± 0.01	101.0	0.67	32.92 ± 0.01	100.0	0.68
Urate oxidase	31.63 ± 0.01	92.0	0.90	31.60 ± 0.01	92.6	0.90	31.56 ± 0.01	89.1	0.96
Xylanase	15.85 ± 0.01	51.0	0.94	15.84 ± 0.01	51.6	0.89	15.87 ± 0.01	50.5	0.92
Lysozyme	14.46 ± 0.01	48.0	0.82	14.46 ± 0.01	46.9	0.90	14.97 ± 0.02	51.3	0.78
RNase A	15.04 ± 0.01	49.0	0.87	15.09 ± 0.01	49.5	0.90	15.08 ± 0.01	48.9	0.92

**Table 4:** Parameters from fits to updated consensus profiles for models with implicit hydration layers χ^2^ values calculated with *σ*_expt_(*q_i_*) weighting. *r*_Sc_ and *c*_1_ are scaling factors for atomic radii that effectively adjust the total excluded volume. *CRYSOL* (version 3.2.1) reports *r*_Sc_ as ‘Adjusted *r*_0_’ whereas *Pepsi-SAXS* reports minimum, maximum and best *r*_0_ values and *r*_Sc_ is calculated as [best *r*_0_/(maximum *r*_0_/1.05)]. %*δρ* is the hydration layer contrast expressed as a percentage of the bulk solvent density. In *FoXS*, *c*_2_ is related to %δρ. *C* is the constant adjustment for the model after scaling to experiment.

	*CRYSOL* Upper set: directional Lower set: dummy waters				*Pepsi-SAXS*				*FoXS*			
	χ^2^	*r*_Sc_	%δρ	*C*	χ^2^	*r*_Sc_	%*δρ*	*C*	χ^2^	*c*_1_	*c*_2_	*C*
Xylose isomerase	652	1.094	0	1.4 × 10^−4^	165	1.03	2.10	−3.2 × 10^−4^	149	1.03	−0.19	−4.2 × 10^−4^
	652	1.094	0	1.4 × 10^−4^								
Urate oxidase	89.6	1.092	3.38	2.2 × 10^−4^	53.3	1.026	3.40	−3.0 × 10^−4^	34.8	1.02	0.74	−6.8 × 10^−4^
	49.7	1.068	4.62	5.7 × 10^−4^								
Xylanase	10.0	1.017	1.45	−4.1 × 10^−3^	6.5	1.015	4.18	3.2 × 10^−3^	4.7	1.02	−0.84	4.8 × 10^−3^
	10.9	1.017	2.36	−3.8 × 10^−3^								
Lysozyme	6.1	1.069	0.90	−4.6 × 10^−3^	7.7	1.022	5.92	4.7 × 10^−3^	10.9	1.02	−0.49	2.8 × 10^−3^
	6.2	1.069	1.34	−4.4 × 10^−3^								
RNase A	175	1.024	3.77	−3.7 × 10^−4^	78.8	1.017	10.0	1.8 × 10^−3^	210	1.01	0.82	−9.0 × 10^−4^
	202	1.028	5.04	4.6 × 10^−5^								

**Table 5:** Parameters from fitting the xylose isomerase tetramer (XI_1_) to pseudo-experimental data of mixtures of XI_1_ with increasing proportions of an arbitrary dimer (XI_2_) The parameters are defined in Table 4 ▸.

	*CRYSOL*				*Pepsi-SAXS*				*FoXS*			
XI_1_:XI_2_ mix	χ^2^	*r*_Sc_	%δρ	*C*	χ^2^	*r*_Sc_	%δρ	*C*	χ^2^	*c*_1_	*c*_2_	*C*
100:0	0.99	1.00	10	9.59 × 10^−6^	1.02	1.00	10	−1.00 × 10^−6^	1.07	1.00	−0.02	1.80 × 10^−5^
98:1	2.36	1.00	10	1.80 × 10^−5^	9.39	1.00	10	−6.70 × 10^−7^	2.46	1.00	0.20	3.70 × 10^−6^
96:2	6.70	0.99	11	2.59 × 10^−5^	14.4	1.01	10	4.39 × 10^−5^	6.93	1.01	0.34	3.16 × 10^−5^
95:2.5	10.1	0.99	12	2.96 × 10^−5^	18.3	1.01	10	6.13 × 10^−5^	10.4	1.00	0.46	2.31 × 10^−5^
94:3	15.5	0.98	13	4.15 × 10^−5^	23.1	1.01	10	7.97 × 10^−5^	14.8	1.01	0.53	3.54 × 10^−5^
92:4	25.6	0.99	14	4.00 × 10^−5^	35.9	1.02	10	1.20 × 10^−4^	26.6	1.00	0.83	7.70 × 10^−6^
90:5	40.8	0.99	14	4.64 × 10^−5^	53.2	1.02	10	1.66 × 10^−4^	42.1	1.01	0.85	6.55 × 10^−5^
80:10	189	0.99	21	7.35 × 10^−5^	222	1.04	10	4.17 × 10^−4^	194	1.02	1.65	1.39 × 10^−4^

**Table 6:** Parameters from fits to updated consensus profiles for *WAXSiS* models Website *WAXSiS* calculations were performed with the ‘thorough’ mode selected for greater convergence. χ^2^ values are calculated with the weighting √(σ_w_^2^ + σ_expt_^2^) using the *Compare* operation within *PRIMUS/Qt*. *WAXSiS* standardly scales experimental data to the model, but here we report the constant adjustment, *C*, after scaling the model to the consensus data for consistency with *CRYSOL*, *Pepsi-SAXS* and *FoXS* (Table 4 ▸).

	Website *WAXSiS*				Custom *WAXSiS*				Mean error ratio
Protein	χ^2^	*C*	Simulation time (ps)	No. of frames averaged	χ^2^	*C*	Simulation time (ps)	No. of frames averaged	Custom *WAXSiS*/website *WAXSiS*
Xylose isomerase	19.0	9.30 × 10^−4^	135.5	259	61.9	9.8 × 10^−4^	50000	5000	0.20
Urate oxidase	5.2	9.0 × 10^−4^	158	300	23.6	1.1 × 10^−3^	50000	5000	0.24
Xylanase	6.6	−1.72 × 10^−3^	541	1042	6.2	−3.1 × 10^−4^	50000	5000	0.29
Lysozyme	6.7	−4.4 × 10^−4^	620.5	1201	11.4	2.2 × 10^−4^	50000	5000	0.39
RNase A	33.0	8.1 × 10^−4^	690.5	1341	56.7	−8.4 × 10^−5^	50000	5000	0.42
