# Supplementary material for: Benchmarking predictive methods for small-angle X-ray scattering from atomic coordinates of proteins using maximum likelihood consensus data
Source: IUCrJ. 2024 Jul 10;11(Pt 5):762–79. doi: 10.1107/S205225252400486X (PMC11364021; doi:10.1107/S205225252400486X)
Supplement: Supplementary file 1 [file m-11-00762-sup1.pdf]

IUCrJ

Volume 11 (2024)

Supporting information for article:

Benchmarking predictive methods for small-angle X-ray scattering from atomic coordinates of proteins using maximum-likelihood consensus data

Jill Trewhella, Patrice Vachette and Andreas Haahr Larsen

Supporting Information

Benchmarking Predictive Methods for Small Angle X-ray Scattering from Atomic Coordinates of Proteins using Maximum Likelihood Consensus Data

Jill Trewhella[a]*, Patrice Vachette[b]* and Andreas Haahr Larsen[c]

[a]School of Life and Environmental Sciences, The University of Sydney, NSW, 2006, Australia

[b]Institute for Integrative Biology of the Cell (12BC) Integrative Biology of the Cell (I2BC), Université Paris-Saclay, CEA, CNRS, Paris, Gif-sur-Yvette, 91198, France

[c]Department of Neuroscience, University of Copenhagen, Blegdamsvej 3, Copenhagen, 2200, Denmark

*Correspondence email: Jill.Trewhella@sydney.edu.au; patrice.vachette@i2bc.paris-saclay.fr

IMPORTANT: this document contains embedded data - to preserve data integrity, please ensure where possible that the IUCr Word tools (available from http://journals.iucr.org/services/docxtemplate/) are installed when editing this document.

Figure S1 Consensus $I(q)$ vs q profiles obtained using *ML-SAScombine* or *DATCOMBINE* (right axes, symbols) with their associated errors (left axes, lines). The combined data for each protein were re-grid in q to approximately the same number of bins to facilitate direct comparison of error magnitudes, and the number of bins in the region 0.08 – 0.5 Å^{-1} is the same in all cases. The colour code designated in panel (A) applies to all.

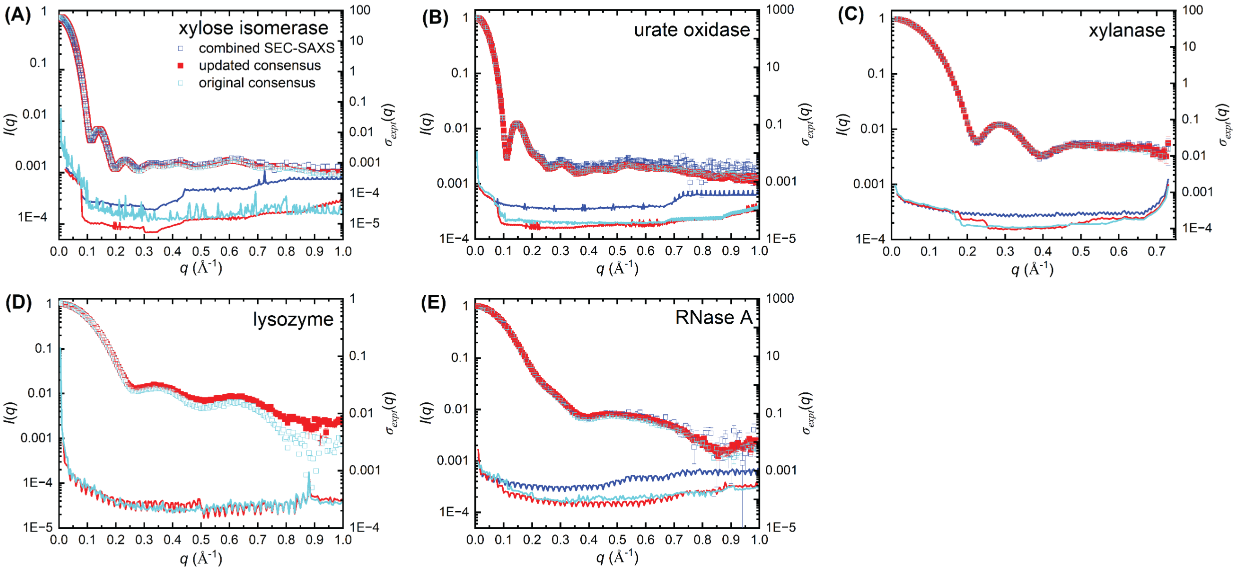

Figure S2 Updated urate oxidase consensus $I(q)$ vs q profile with fitted model profiles (left axes) and corresponding error weighted residual difference plots below (right axes) from *CRYSOL* using either the directional (classic) hydration layer model or dummy waters.

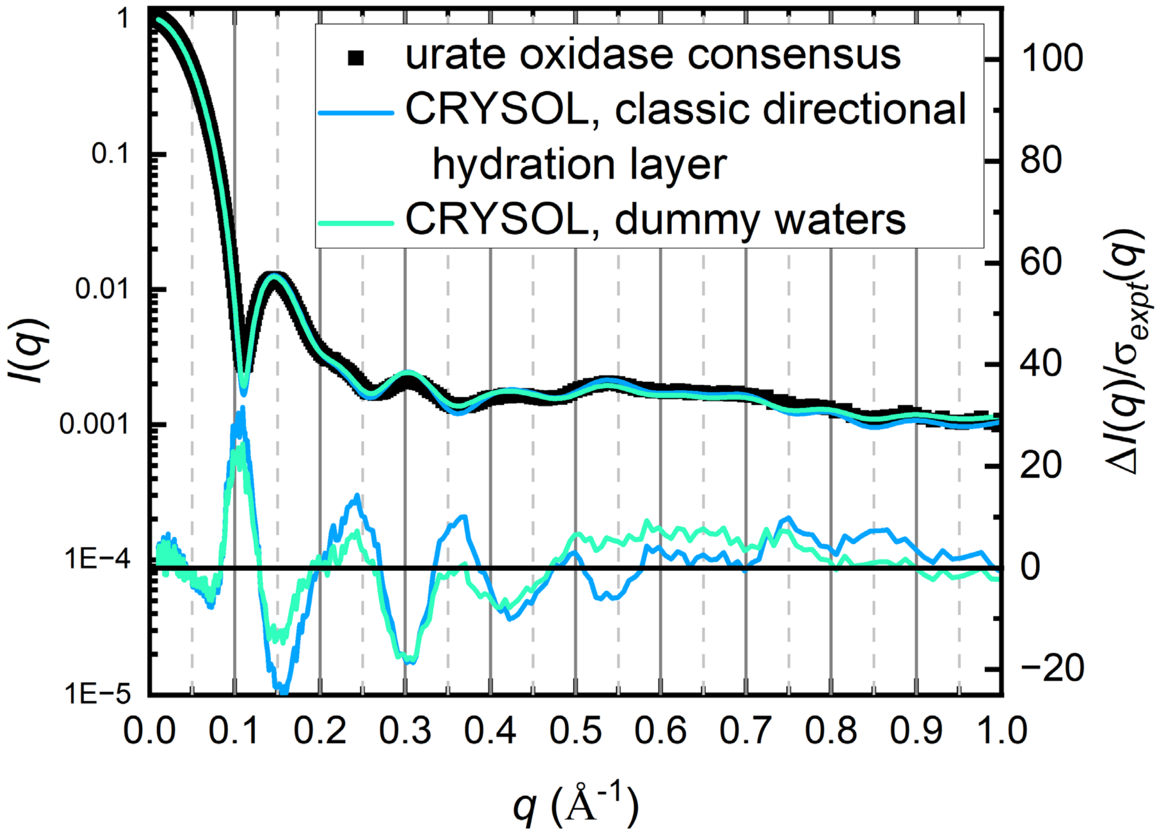

Figure S3 Updated consensus $I(q)$ vs q profiles with fitted model profiles (left axes) and corresponding error weighted residual difference plots below (right axes). To facilitate direct comparison, the custom *WAXSiS* residual differences are calculated with experimental statistical error weighting only (*i.e.* *WAXSiS* statistical errors, σ_w, set to zero) and the consensus data have been re-gridded to a common q-grid with the custom *WAXSiS*. The horizontal dashed lines indicate $\Delta I(q)/\sigma_{\mathrm{expt}}(q) = \pm 3$. The colour code in panel (A) is used for all.

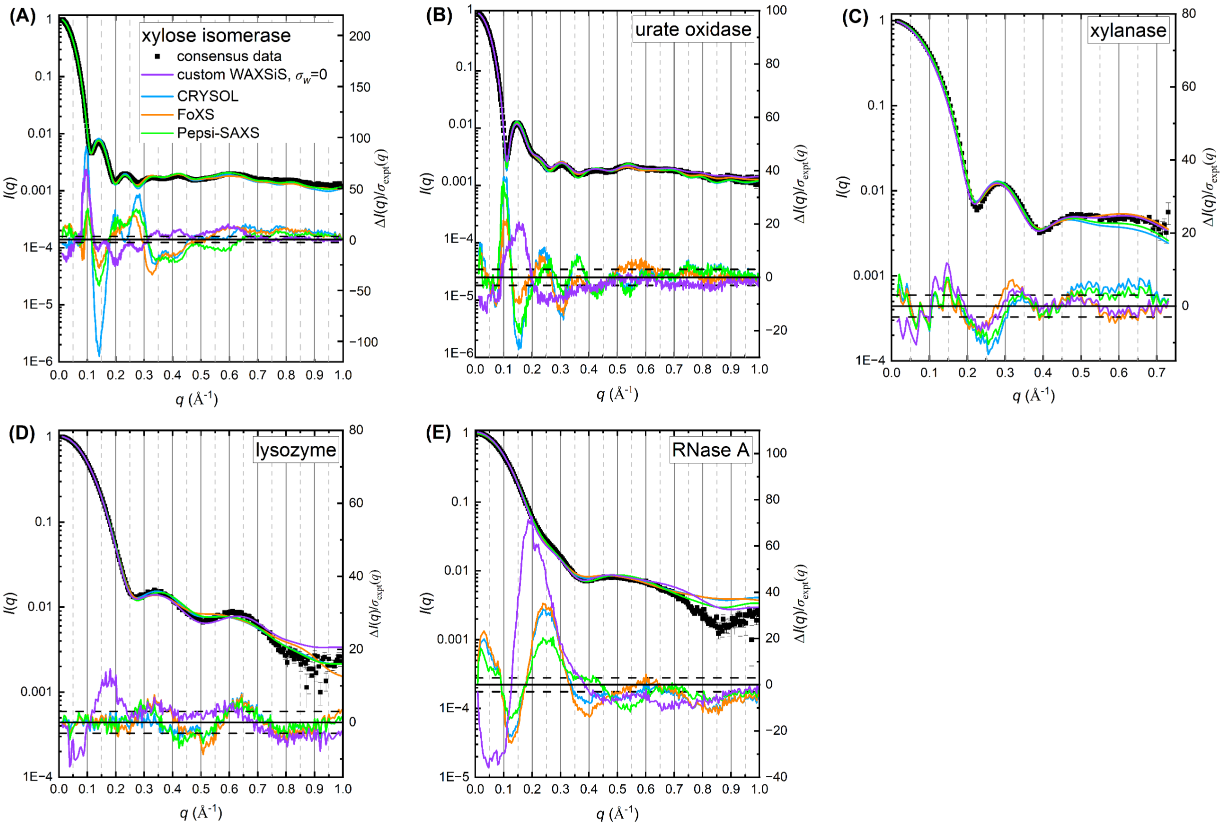

Figure S4 *WAXSiS* statistical errors, $\sigma_w^2(q)$ vs q (left axis, filled symbols) and the ratio $\sigma_w(q)/\sigma_{expt}(q)$ vs q (right axis, empty symbols) for the website *WAXSiS* predicted profile with the thorough mode selected (100s of ps simulations) (blue) and the custom *WAXSiS* profile (50 ns simulations) (red). Right axis horizontal grid lines are shown with $\sigma_w(q)/\sigma_{expt}(q) = 1$ as a solid grey line.

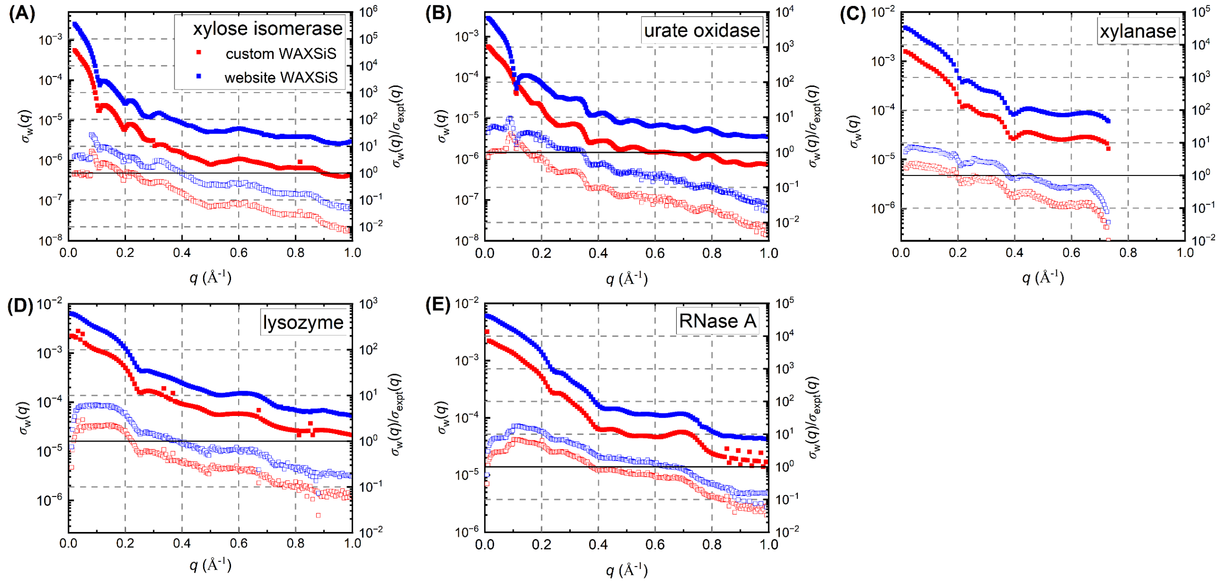

Table S1 Parameters from fits to updated consensus profiles after re-gridding for direct comparison with the custom *WAXSiS* calculations from the round robin study for xylose isomerase, urate oxidase, xylanase, lysozyme, and RNase A.

Program	*WAXSiS* Upper set: custom Lower set: Website χ^2 with error weighting, $\sigma_{expt}(q_i)$, only		*CRYSOL* Upper set: directional Lower set: dummy waters				Pepsi-SAXS				*FoXS*			
Protein	χ^2	*C*	χ^2	r_{Sc}	% δρ	*C*	χ^2	r_{Sc}	% δρ	*C*	χ^2	c_2	c_2	*C*
Xylose isomerase	228	9.8E-4	949	1.088	0	3.8E-4	227	1.027	1.40	-4.9E-4	165	1.03	-0.32	-6.3E-4
	1250	9.3E-4	949	1.088	0	3.8E-4								
Urate oxidase	38.2	1.1E-3	74.4	1.094	2.74	3.3E-4	51.8	1.027	2.90	-4.0E-4	27.3	1.02	0.52	-8.1E-4
	61.5	9.0E-4	45.2	1.074	3.72	6.5E-4								
Xylanase	15.2	-3.1E-4	24.7	1.023	1.44	-1.9E-3	15.6	1.015	4.30	2.6E-3	11.2	1.03	-0.85	2.6E-3
	72.0	-1.6E-3	27.0	1.017	2.34	-1.6E-3								
Lysozyme	22.9	2.2E-4	5.8	1.071	0.85	-3.8E-3	7.3	1.024	5.90	4.0E-3	10.2	1.02	-0.51	2.0E-3
	22.6	-1.1E-3	5.9	1.071	1.26	-3.7E-3								
RNase A	551	-8.4E-5	140	1.024	3.75	4.3E-4	63.1	1.016	10.0	1.3E-3	169	1.01	0.82	-1.4E-3
	558	-3.9E-4	163	1.029	5.02	5.2E-4								

χ^2 values with $\sigma_{expt}(q_i)$ weights are as reported by each method except for *WAXSiS* values that were calculated using the Compare function in *PRIMUS/Qt*. r_{Sc} and c_1 are scaling factors for atomic radii that effectively adjust the total excluded volume by r_{Sc}^3. %$\delta\rho$ is the hydration layer contrast expressed as a percent of the bulk solvent density. *C* is the constant adjustment for the model to experiment. For *FoXS*, c_2 is related to %$\delta\rho$ (see main text 1.1). *WAXSiS* scales experiment data to model, and here we report the constant adjustment for fitting the model scaled to experiment for consistency with the other methods.

Table S2 Guinier R_g values (in Å) for the updated consensus profiles compared to values obtained by Guinier analysis of the various model fits.

Protein	Consensus	*CRYSOL*	*FoXS*	*Pepsi-SAXS*	website *WAXSiS*	custom *WAXSiS*
Xylose isomerase	33. 15±0.03	33.09	33.08	33.07	33.12	33.20
Urate oxidase	32.22±0.03	31.91	31.97	31.92	31.76	32.05
Xylanase	16.15±0.01	16.00	16.02	15.96	15.96	16.07
Lysozyme	14.54±0.01	14.52	14.56	14.52	14.58	14.59
RNase A	15.11±0.01	14.88	14.85	14.92	14.69	15.09
